# Kilowatt-class planar four-way gysel combiner with power-aware impedance optimization for L-band pulsed radar

**DOI:** 10.1371/journal.pone.0354086

**Published:** 2026-07-24

**Authors:** Mohamed Mowafy, Mahmoud A. Shawky, Syed Tariq Shah, Abdelhamid A. Shaalan, Safaa Ibrahim, Mai F. Ahmed

**Affiliations:** 1 Department of Electronics and Communication Engineering, Faculty of Engineering, Zagazig University, Zagazig, Egypt; 2 The Egyptian Technical Research and Development Center, Cairo, Egypt; 3 School of Computer Science and Electronic Engineering, University of Essex, Colchester, United Kingdom; Southern University of Science and Technology, CHINA

## Abstract

This work presents a compact planar four-way *Gysel* power divider/combiner designed for kilowatt-class coherent power combining in L-band pulsed radar transmitters. Unlike previously reported waveguide and coaxial Gysel architectures, the proposed planar implementation achieves a 3 kW peak output on an RO4003C substrate while maintaining low-loss, highly manufacturable microstrip construction. A power-aware full-wave electromagnetic optimization framework is introduced to refine characteristic impedances and electrical lengths, explicitly accounting for current distribution, thermal dissipation, and fabrication tolerance sensitivity, resulting in improved mid-band isolation and stable high-power performance. The fabricated prototype achieves an input return loss better than 15 dB, inter-port isolation exceeding 25 dB, amplitude imbalance within ±0.3 dB, and phase deviation within $\pm 1^{\circ }$ across 1.2–1.4 GHz. Grounded isolation branches equipped with flange-mounted high-power resistors, together with thermally optimized via arrays, ensure efficient dissipation of odd-mode energy under mismatch conditions. Experimental validation shows close agreement with simulation results, demonstrating that the proposed design represents a rare example of a planar microstrip Gysel combiner experimentally validated for reliable kilowatt-class operation in L-band solid-state radar systems, achieving a practical balance between power handling, compactness, and manufacturability.

## 1. Introduction

Modern solid-state radar transmitters increasingly employ the parallel operation of multiple medium-power amplifier modules (PAMs) to achieve kilowatt-class peak output power while maintaining high reliability and scalability. By distributing the required output power across several PAMs, modularity and fault tolerance are enhanced; however, this architecture imposes stringent requirements on the power-combining network, which must sustain high combining efficiency, preserve phase coherence among channels, and provide robust thermal performance under mismatch conditions and pulsed high-power operation. In this context, the *Gysel* power divider/combiner has emerged as a suitable topology for high-power RF applications. Unlike the Wilkinson structure, which employs floating isolation resistors, the *Gysel* topology relocates the isolation loads to grounded branches, thereby enabling a direct thermal path to the chassis and significantly improving heat dissipation and fault tolerance under high-voltage standing wave ratio (VSWR) conditions [1,2]. As a result, the *Gysel* architecture is particularly well suited for L-band pulsed radar transmitters, where high peak power levels and limited thermal margins demand efficient heat extraction to prevent device degradation. Compared to waveguide-based Gysel combiners, which typically achieve very low insertion loss and superior thermal margins at the expense of increased size, weight, and fabrication complexity, planar microstrip implementations offer a compact and manufacturable alternative, albeit with more stringent design constraints regarding loss and heat dissipation. This paper presents the design, full-wave electromagnetic optimization, fabrication, and experimental validation of a planar four-way *Gysel* power combiner implemented on an RO4003C substrate for L-band pulsed radar applications. The proposed design employs a simulation-driven impedance optimization methodology together with thermally optimized layout techniques and grounded isolation branches to achieve reliable kilowatt-class performance. In contrast to many previously reported planar dividers that are typically limited to sub-kilowatt operation, the present work targets multi-kilowatt pulsed performance while preserving compactness and manufacturability. Experimental results confirm that the developed network maintains low input reflection, high inter-port isolation, accurate amplitude balance, and tight phase coherence, as required for efficient coherent power combining.

### 1.1. Contributions

The main contributions of this work can be summarized as follows:

1. Design and fabrication of a planar high-power four-way *Gysel* divider/combiner specifically tailored for L-band pulsed radar transmitters.
2. Simulation-driven full-wave electromagnetic optimization and experimental validation demonstrating high inter-port isolation, improved impedance matching, accurate amplitude balance, and phase coherence suitable for multi-kilowatt coherent power combining.
3. Integration of practical high-power design features, including grounded isolation branches, flange-mounted high-power resistors, and dense ground-via carpets, to enhance thermal robustness and fault tolerance under pulsed operation.
4. A systematic comparison with reported planar and waveguide power combiners is provided to position the proposed design in terms of power handling, insertion loss, and implementation trade-offs.

### 1.2. Literature review

Power dividers and combiners are fundamental building blocks in RF and microwave systems, enabling signal distribution, coherent power combining, and phased-array beamforming. Classical architectures such as the Wilkinson divider [3] offer good isolation and excellent matching; however, their reliance on floating isolation resistors limits heat extraction and reduces suitability for high-power operation. In contrast, the *Gysel* divider [1] relocates the isolation resistors to grounded branches, enabling direct heat sinking, improved fault tolerance under mismatch conditions, and enhanced power-handling capability. These advantages have established the *Gysel* topology as a preferred solution for high-power radar and communication transmitters.

Recent advances in PCB-integrated aerospace systems further highlight the importance of compact planar implementation and effective thermal management. For instance, Khan *et al.* [4] presented PCB-integrated embedded planar magnetorquers for nanosatellite applications, where high integration density and thermal constraints are critical design considerations. These systems utilize multilayer PCB structures with embedded active elements, demonstrating efficient space utilization and thermally constrained operation. Such challenges are closely aligned with those encountered in high-power planar RF combiners, emphasizing the importance of advanced PCB-based integration techniques in achieving reliable, compact, and high-performance electromagnetic systems.

Over the past two decades, extensive research has advanced *Gysel*-based architectures along several major directions:

1. *Bandwidth Enhancement:* Numerous studies have proposed modified *Gysel* structures employing stepped-impedance sections, multilayer implementations, and compensation networks to extend operational bandwidth. Fractional bandwidths exceeding 60% have been demonstrated using hybrid transmission-line sections and substrate stacking techniques [5–7]. These approaches primarily target wideband communication systems and generally do not address the thermal and power-handling requirements of kilowatt-class radar applications.
2. *High-Power Handling:* To address power limitations, waveguide-based *Gysel* combiners, including ridge waveguide and rectangular coaxial configurations, have been reported [8,9]. These implementations significantly improve conduction cooling, mitigate current crowding, and increase breakdown thresholds, making them suitable for multi-kilowatt continuous-wave operation. These structures typically achieve insertion losses below 0.2 dB and support power levels beyond several kilowatts, but at the expense of significantly larger volume, higher fabrication cost, and reduced compatibility with planar integration. Nevertheless, their increased size, cost, and fabrication complexity restrict their applicability in compact solid-state radar transmitters.
3. *Hybrid and Filtering Power Dividers:* Hybrid Wilkinson-*Gysel* architectures and filtering power combiners have been introduced to reduce size, weight, and power (SWaP) while improving spectral selectivity [10,11]. By integrating power division, isolation, and filtering functions, these designs enable compact front-end modules but typically sacrifice peak power capability or thermal robustness.
4. *Miniaturization and Integration:* Compact implementations based on HMSIW, LTCC, and substrate-integrated technologies have demonstrated dual-band and ultra-wideband performance [12]. While these techniques offer high integration density, their limited thermal paths and dielectric breakdown constraints render them unsuitable for kilowatt-class radar transmitters.

Furthermore, recent work on PCB-integrated electromagnetic subsystems for space-constrained platforms, such as reconfigurable embedded planar structures for nanosatellite applications, has demonstrated the feasibility of integrating high-reliability RF functions within compact planar substrates. These approaches emphasize the importance of thermal management, structural robustness, and integration density, challenges that are directly relevant to kilowatt-class planar power combiners.

Despite these advances, achieving *kilowatt-class* performance using a planar microstrip topology remains a significant challenge. High-power planar dividers are constrained by conductor heating, dielectric losses, and inefficient thermal extraction through standard PCB substrates. In addition, the impedance ratios required for ideal *Gysel* operation become increasingly sensitive to fabrication tolerances at elevated power densities.

#### 1.2.1. High-power handling and thermal management.

At power levels approaching several kilowatts, robustness under mismatch conditions, thermal efficiency, and fault tolerance become dominant design considerations. The *Gysel* topology inherently addresses these requirements by employing grounded isolation resistors, which provide a direct thermal path to the chassis and offer a clear advantage over Wilkinson structures, where floating resistors are prone to localized overheating [1,3]. While waveguide and coaxial *Gysel* implementations demonstrate excellent thermal margins [8,9], they typically involve significantly larger physical footprints and higher material and machining costs compared to planar PCB-based approaches.

For planar implementations, recent studies have shown that enhanced power handling can be achieved through impedance re-engineering near the combining junction, dense ground-via arrays, and optimized thermal spreading structures [13]. Nonetheless, simultaneously maintaining low insertion loss, high inter-port isolation, and tight phase balance remains nontrivial, particularly for L-band pulsed radar systems that demand both high peak power and precise phase coherence. The present work builds on these insights by adopting a classical *Gysel* framework while introducing power-aware impedance optimization, grounded thermal paths, and layout strategies specifically tailored for kilowatt-class L-band pulsed radar transmitters.

#### 1.2.2. Hybrid and arbitrary split designs.

Recent investigations have explored hybrid architectures that combine the complementary characteristics of Wilkinson and *Gysel* dividers, enabling trade-offs among bandwidth, isolation, and power-handling capability [14]. Such structures typically exploit the Wilkinson divider’s strong in-band isolation together with the superior thermal path of the *Gysel* topology. In parallel, modified *Gysel* configurations have been proposed to realize arbitrary power division ratios, real terminated impedances, and improved matching performance [15,16]. Additional developments include generalized broadband asymmetrical Wilkinson dividers, T-coil-loaded ultra-wideband structures, and miniaturized layouts targeting footprint reduction in highly integrated RF front ends [17–19]. Although these approaches demonstrate significant flexibility, they typically prioritize bandwidth or size over kilowatt-class power robustness.

#### 1.2.3. Design foundations and CAD guidance.

Microstrip implementations of *Gysel* and Wilkinson dividers commonly begin with closed-form approximations for characteristic impedance and electrical length, with the Hammerstad-Jensen formulation serving as a widely adopted synthesis model for initial line-dimension estimation [20]. Design tables and CAD-oriented guidelines further facilitate the realization of multi-way networks by providing scalable impedance and quarter-wave transformation rules for various division ratios [21]. Foundational microwave engineering texts, such as [2], remain essential for understanding even- and odd-mode operation, power-splitting symmetry, and isolation mechanisms. Complementary treatments of transmission-line theory and microstrip implementation constraints are provided in classical monographs [22], forming the analytical basis for both hand calculations and full-wave optimization in modern RF CAD workflows.

#### 1.2.4. Rationale for this work.

A recurring theme in prior research is the fundamental trade-off among operational bandwidth, structural simplicity, thermal robustness, and peak power capability. Many bandwidth-enhanced or miniaturized divider/combiner architectures compromise thermal paths or rely on high-impedance features that are unsuitable for kilowatt-level operation. In contrast, waveguide- and coaxial-based implementations achieve superior power handling and thermal robustness; however, these approaches typically incur increased size, higher fabrication complexity, and limited compatibility with planar integration.

To further elucidate these trade-offs, a quantitative comparison of representative planar, coaxial, and waveguide Gysel-based implementations is presented in Table 1. The comparison includes key performance metrics such as operating frequency, power handling capability (expressed in equivalent CW values where applicable), isolation, phase imbalance, and insertion loss. This enables a direct and fair assessment of the advantages and limitations of each implementation technology and clearly positions the proposed work within the current state-of-the-art.

**Table 1:** Comparison with recent Gysel-based high-power dividers/combiners.

Ref	Year	Freq (GHz)	Topology	Power (W, CW)	ISO (dB)	PI	IL	Remarks
[5]	2013	4–8	Planar Gysel	∼50–70	∼20	±1	∼1–1.5	2-Way wideband planar
[8]	2019	3–5	Ridge-waveguide Gysel	∼5000	15–25	±1–2	∼0.1–0.2	2-Way high-power, low-loss
[9]	2015	4–5.5	Rectangular coaxial waveguide	1000–2000	5–15	±5	∼0.7	4-way chained quasi-planar
[11]	2025	4.5–5.5	Filtering Gysel	∼50	20–30	±1	∼0.2	Integrated filtering functionality
[13]	2022	2.3–2.5	Planar APHC Gysel	∼100	20–25	±3–5	∼0.1–0.2	2-Way thermal-aware design
[23]	2025	0.38	Modified Gysel	∼500	∼23	±1–3	∼0.16	2-Way compact high-power
**This work**	2026	1.1–1.5	Planar RO4003C	∼300	>25	±1	∼0.4	4-Way,3 kW peak

Motivated by these limitations, this work adopts the classical *Gysel* framework while introducing power-aware impedance optimization, grounded isolation loading, and thermally optimized planar layout strategies. The objective is to realize a compact planar microstrip combiner capable of delivering robust and repeatable kilowatt-class performance at L-band, while maintaining coherent power combining without sacrificing thermal reliability or manufacturability.

As shown in Table 1, waveguide and coaxial implementations achieve superior power handling capability and lower insertion loss due to their inherently higher thermal margins and reduced conductor losses. However, these approaches are typically associated with larger physical size, increased fabrication complexity, and reduced integration flexibility. In contrast, planar implementations provide advantages in compactness, manufacturability, and system-level integration, albeit with moderate compromises in loss and power density. The proposed design achieves a balanced trade-off by enabling kilowatt-class operation within a compact planar microstrip architecture, while maintaining competitive isolation, phase coherence, and acceptable insertion loss for high-power L-band applications.

## 2. Design methodology

This section discusses the detailed description of the proposed work.

### 2.1. Specifications and substrate

The system reference impedance is fixed at 50 Ω. Four power amplifier modules (PAMs), each delivering 800 W under 300 µs pulsed operation at a 10% duty cycle with a + 50 V drain bias, are coherently combined to achieve a total peak output power of 3 kW. The target operating band spans 1.2–1.4 GHz, while extended characterization is performed over 1.1–1.5 GHz to evaluate off-nominal and wideband behavior. The RO4003C substrate is selected for its stable dielectric constant, low loss tangent, and demonstrated suitability for high-power planar microstrip implementations at L-band.

### 2.2. Design approach and impedance optimization

Unlike conventional *Gysel* implementations based on closed-form synthesis, this work adopts a fully simulation-driven design methodology using CST Studio Suite. The initial layout follows the canonical *Gysel* topology, with all microstrip sections designed to be quarter-wavelength (λ/4) at a center frequency of 1.3 GHz. Starting from this baseline, the characteristic impedances *Z*_1_, *Z*_2_, *Z*_3_, and *Z*_4_ are iteratively optimized through full-wave electromagnetic simulations to satisfy the following performance requirements:

- input return loss ≥15 dB over 1.2–1.4 GHz,
- inter-port isolation ≥25 dB,
- amplitude imbalance within ±0.3 dB,
- phase deviation within $\pm 1^{\circ }$.

In this work, the optimization process is explicitly constrained by high-power operation considerations, leading to what is referred to as a “power-aware” design approach. In addition to standard electromagnetic objectives such as matching, isolation, and phase balance, the optimization incorporates practical high-power constraints including current density distribution, thermal dissipation paths, and sensitivity to fabrication tolerances. These additional constraints guide the selection of characteristic impedances and physical dimensions toward configurations that reduce localized current crowding, improve heat spreading through grounded vias, and enhance robustness under mismatch and pulsed excitation conditions. As a result, the optimization process extends beyond conventional S-parameter tuning and targets reliable operation under kilowatt-class power levels.

**Fig 1:**
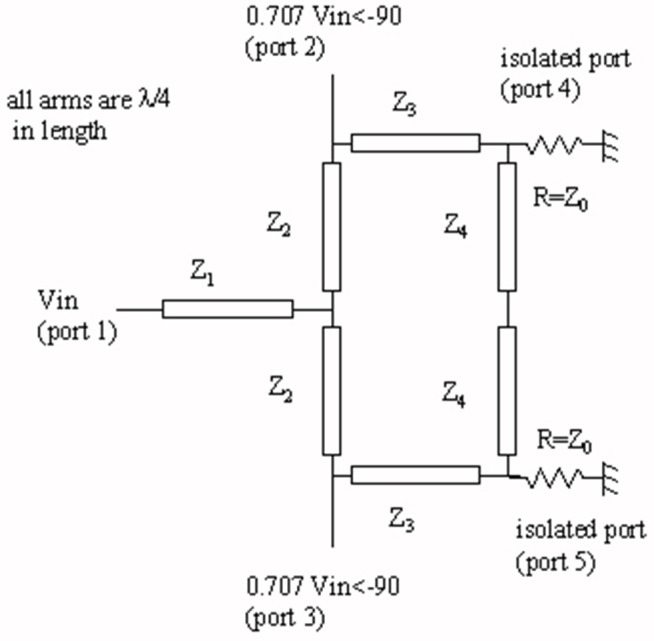
Modified equal-split *Gysel* topology optimized in CST Studio Suite. The input section *Z*_1_ is fixed at 50 Ω, while the remaining impedances (*Z*_2_, *Z*_3_, *Z*_4_) are optimized for high-power operation. Isolation resistors (*R* = *Z*_0_) are implemented using flange-mounted high-power devices to improve thermal performance.

**Fig 2:**
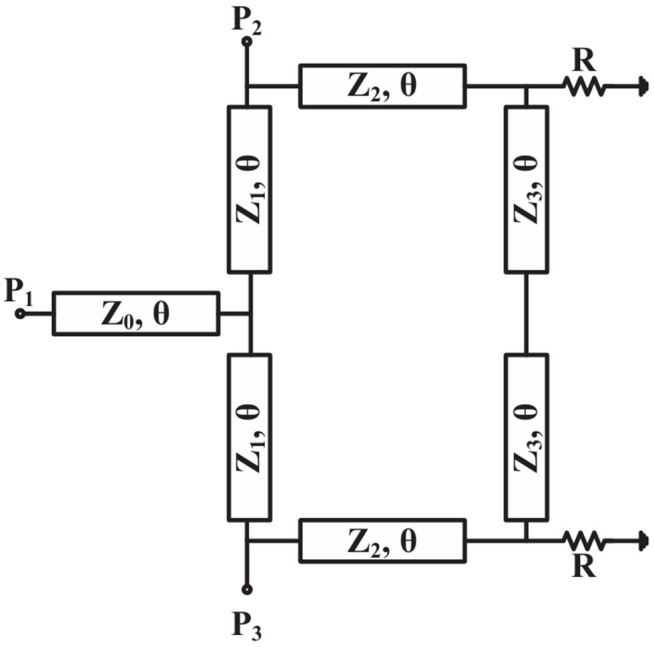
Composite even/odd-mode equivalent circuits for the two-way *Gysel* divider showing impedances $Z_{1},Z_{2},Z_{3},Z_{4}$ and isolation resistor *R.* These diagrams provide physical insight into power division, impedance transformation, and isolation behavior.

To simplify fabrication, reduce conductor loss, and mitigate sensitivity to manufacturing tolerances, the input section impedance *Z*_1_ is fixed at 50 Ω. The remaining impedances are subsequently tuned to achieve the required electrical performance while maintaining practical line widths and physical spacing, see Figs 1 and 2. The resulting optimized values are:


$$\begin{array}{c} Z_{1}=50\;\Omega ,\;Z_{2}=75\;\Omega ,\;Z_{3}=50\;\Omega ,\;Z_{4}=30\;\Omega ,\;. \end{array}$$

*Bandwidth-Isolation Trade-off* Departing from the canonical *Gysel* impedance ratios increases the contrast between *Z*_2_ and *Z*_4_, thereby enhancing odd-mode energy absorption and improving mid-band isolation. This enhancement, however, introduces increased frequency sensitivity in the impedance transformation, resulting in a reduced operational bandwidth. CST’s optimization framework enables a systematic exploration of this trade-off, allowing selection of an impedance set that balances bandwidth, isolation, and manufacturability for L-band pulsed radar operation. Furthermore, the selected impedance set was verified to maintain stable performance under small geometric perturbations, which are representative of practical fabrication tolerances, and a detailed tolerance analysis is presented in Section 4.3 to quantify the impact of such variations on phase balance, matching, and isolation.

### 2.3. CST-Optimized impedance values

Table 2 summarizes the optimized characteristic impedance values obtained from full-wave CST simulations for the two-way *Gysel* cell.

**Table 2:** Final CST-optimized impedance values for the two-way *Gysel* cell (*Z*_0_ = 50 Ω).

Parameter	Final Design Value
*Z*_1_ (input section)	50 Ω (fixed for manufacturability)
*Z*_2_ (junction section)	75 Ω
*Z*_3_ (output section)	50 Ω
*Z*_4_ (isolation branch)	30 Ω
*R* (isolation resistor)	50 Ω (flange-mounted)

*Note:* All transmission-line sections are designed to be λ/4 at 1.3 GHz. The CST optimization process accounts for parasitic discontinuities, via inductance, dielectric inhomogeneity, and practical fabrication constraints, factors that are critical for achieving reliable kilowatt-class performance in planar RF structures. In addition, the selected impedance configuration has been verified to provide stable electromagnetic behavior under small perturbations in line dimensions and substrate parameters, ensuring robustness against practical fabrication tolerances and process variations.

## 3. Even/Odd-mode analysis

Although the impedance values used in this work were ultimately obtained through CST full-wave optimization, even/odd-mode analysis remains fundamental for understanding the operating principles of the *Gysel* topology. This modal decomposition provides physical insight into how the divider achieves equal power division under symmetrical excitation and high inter-port isolation when ports are excited out of phase. While CST simulations inherently validate these behaviors, the analytical formulation is included here for completeness and conceptual clarity.

### 3.1. Equivalent circuits and symmetry conditions

**Fig 3:**
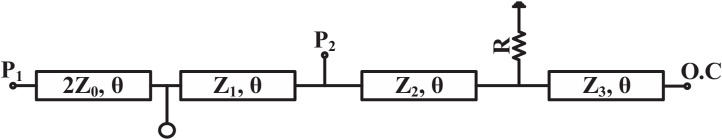
Even-mode equivalent circuit (symmetry plane open). The isolation branch presents an effective open circuit near *f*_0_, and the quarter-wave line sections $\left\{Z_{1},Z_{2},Z_{3},Z_{4}\right\}$ transform impedances to yield equal power split and proper input matching.

**Fig 4:**
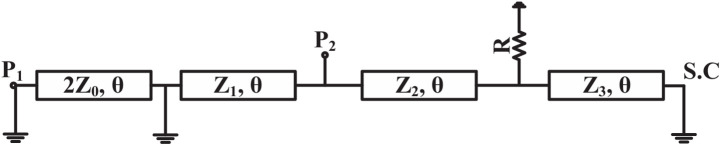
Odd-mode equivalent circuit (symmetry plane short). The grounded isolation resistor, viewed through a λ/4 line of impedance $Z_{s}$, is transformed to $Z_{sh}=Z_{s}^{2}/R$, enabling absorption of anti-phase energy and enforcing high isolation between the output ports.

Figs 3 and 4 illustrate the equivalent circuits corresponding to the even and odd excitation modes. Under even-mode excitation, the symmetry plane behaves as an open circuit (no current flows across the boundary). Conversely, in the odd mode, the symmetry plane becomes a virtual ground (zero voltage), enabling the isolation branch to participate in absorbing imbalance energy.

#### 3.1.1. Odd-mode analysis.

In the odd mode, the symmetry plane behaves as a virtual ground. The grounded isolation resistor is viewed through a quarter-wavelength transmission line of impedance $Z_{s}$, leading to the transformed impedance at the junction:


$$\begin{array}{c} Z_{sh}(f_{0})=\frac{Z_{s}^{2}}{R},\;Y_{sh}(f_{0})=\frac{R}{Z_{s}^{2}}. \end{array}$$ (1)

For classical analytical treatments, the following canonical values are often selected:


$$\begin{array}{c} Z_{t}=\sqrt{2}\;Z_{0},\;Z_{s}=\sqrt{2}\;Z_{0},\;R=Z_{0}, \end{array}$$ (2)

which ensure that the odd-mode junction is matched and that ideal isolation ($S_{23}(f_{0})=0$) is achieved. These choices satisfy the classical *Gysel* design conditions described in foundational microwave theory.

*Note:* These canonical values are presented solely for conceptual understanding. In this work, all characteristic impedances were determined through full-wave CST optimization rather than closed-form synthesis, allowing the design to meet the strict thermal, bandwidth, and power-handling constraints required for kilowatt-class L-band operation. Furthermore, the even/odd-mode framework provides qualitative insight into how deviations in electrical length or impedance values influence matching and isolation, forming the analytical basis for the tolerance analysis discussed in Section 4.3.

### 3.2. Structure and assembly

**Fig 5:**
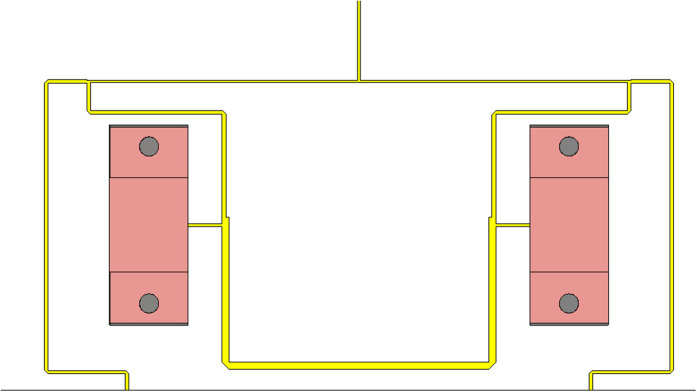
Stage 1: Two-way *Gysel* layout on RO4003C substrate featuring grounded isolation branches to enable efficient heat sinking (simulated using CST Studio Suite).

**Fig 6:**
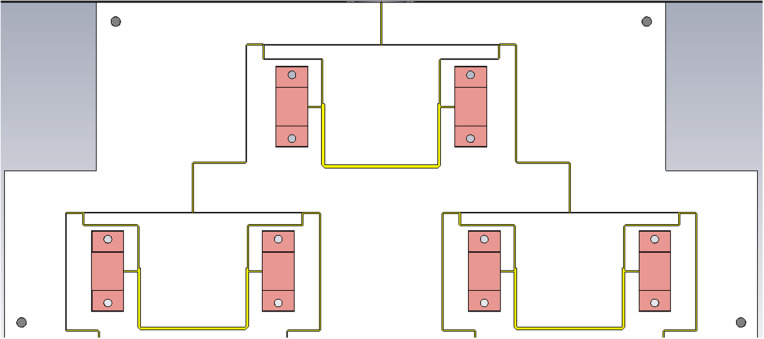
Stage 2: Four-way network created by cascading two two-way cells with equal electrical lengths to ensure coherent four-port operation (simulated using CST Studio Suite).

**Fig 7:**
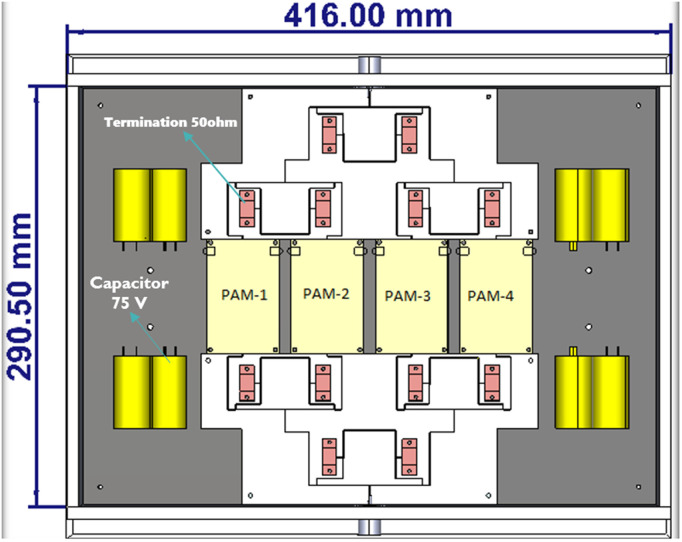
Final system integration showing the splitter, four PAMs, and the four-way combiner inside a compact enclosure with short RF interconnects and wide DC feedlines optimized for pulsed operation (simulated using CST Studio Suite).

The implementation of the proposed design was carried out in two stages to enable progressive validation of performance and to mitigate integration risks. In *Stage 1*, a two-way *Gysel* cell was designed and fabricated to verify impedance matching, inter-port isolation, and power-splitting behavior under moderate power conditions, as shown in Fig 5. In *Stage 2*, the validated two-way cell was cascaded to realize a four-way configuration with equalized electrical paths, enhanced thermal handling, and mechanical integration suitable for kilowatt-class pulsed radar operation, as illustrated in Fig 6. This modular and hierarchical design approach also facilitates scalability, allowing extension to higher-order configurations (e.g., 8-way or 16-way combiners). However, scaling to higher port counts using the same substrate (RO4003C) and identical PAM modules may result in total power levels exceeding the thermal and conductor loss handling capabilities of the planar structure. To address this limitation, practical scaling can be achieved either by reducing the output power per PAM module or by employing high-thermal-conductivity substrates (e.g., ceramic-based materials or metal-backed structures) to enhance power-handling capability. Fig 7 illustrates the final system integration, highlighting the compact arrangement of the splitter, four PAMs, and the four-way combiner with optimized RF interconnects and DC feedlines tailored for pulsed operation. Furthermore, the two-stage validation strategy enables progressive verification of both electromagnetic performance and tolerance robustness. This approach ensures that small deviations in individual unit cells do not accumulate significantly in the final cascaded structure, thereby maintaining phase coherence and combining efficiency in the full system implementation.

## 4. Electromagnetic simulation results

Full-wave frequency-domain simulations were performed in CST Studio Suite across 1.1–1.5 GHz to verify compliance with the design requirements, including input matching, port-to-port isolation, amplitude balance, and phase coherence. The wide simulated band also allows assessment of off-nominal behavior and sensitivity to fabrication tolerances.

### 4.1. Two-way cell performance

**Fig 8:**
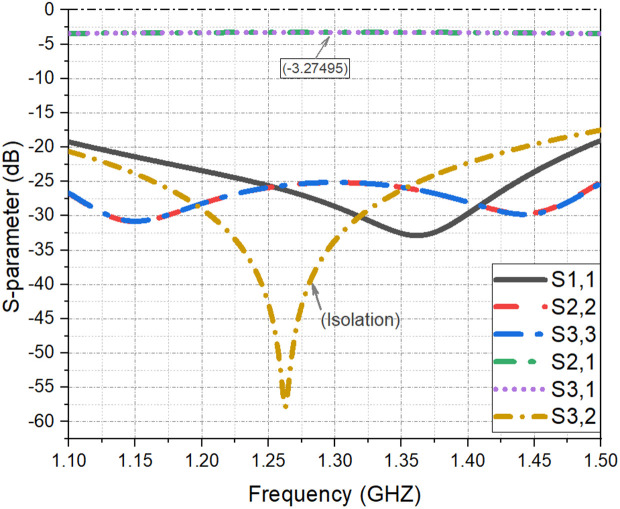
Simulated *S*-parameters of the two-way *Gysel* cell over 1.1-1.5 GHz.

Fig 8 presents the simulated *S*-parameters for the two-way *Gysel* cell. The input return loss ($S_{11}\approx -20\;dB$) demonstrates excellent matching across the band. The transmission coefficients (*S*_21_ and $S_{31}\approx -3.27\;dB$) remain close to the theoretical −3.01 dB ideal, indicating minimal insertion loss. Inter-port isolation exceeds −55 dB, confirming that the grounded isolation branches effectively absorb odd-mode energy, a key requirement for stable high-power combining.

### 4.2. Four-way network: phase and amplitude balance

**Fig 9:**
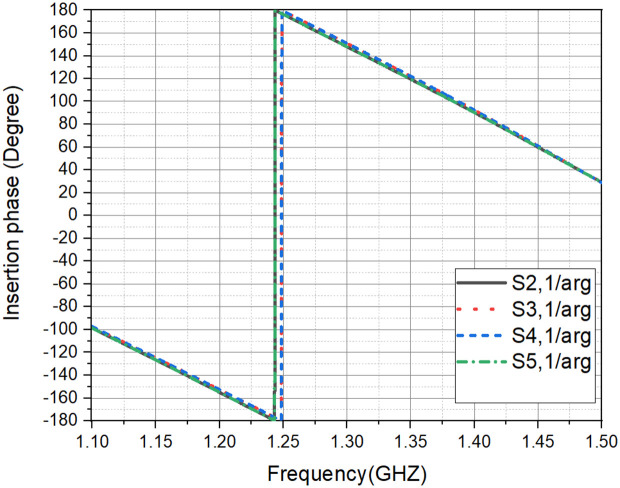
Simulated insertion phase for the four-way network, demonstrating a phase spread within $\pm 1^{\circ }$ at 1.3 GHz.

**Fig 10:**
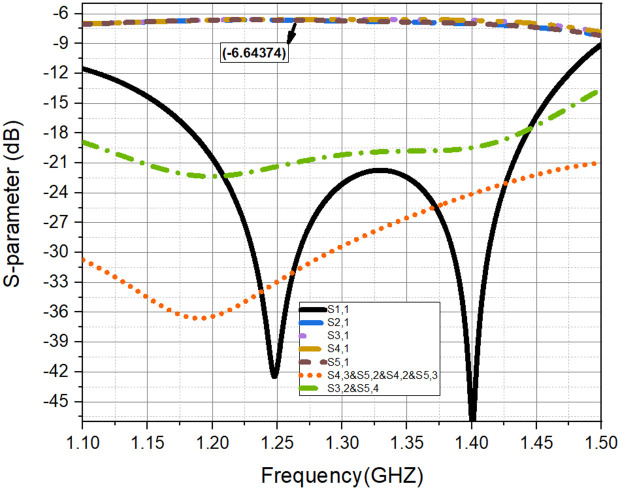
Simulated *S*-parameters for the four-way network showing strong amplitude balance and inter-port isolation.

The four-way network, realized by cascading two-way cells, maintains excellent coherence among the four output paths. As shown in Fig 9, the insertion phase variation across the four ports is within $\pm 1^{\circ }$ at 1.3 GHz, ensuring effective coherent power combining. Amplitude imbalance remains within 0.6 dB of the ideal −6.02 dB split, with the simulated magnitudes $S_{2,1}-S_{5,1}\approx -6.64\;dB$, as illustrated in Fig 10. The four-way structure preserves strong matching at the common port (*S*_11_ > 15 *dB*) and inter-port isolation exceeding −25 dB across the operating band.

### 4.3. Tolerance analysis

To evaluate the robustness of the proposed design under practical fabrication conditions, a tolerance analysis was performed to assess the sensitivity of key performance metrics to variations in transmission-line dimensions and substrate parameters. Manufacturing tolerances in standard PCB fabrication can introduce variations in line length and width, typically on the order of ±2%, as well as dielectric constant deviations ($\Delta \varepsilon_{r}\approx \pm 0.05$ for RO4003C). These variations primarily affect the effective electrical length of the quarter-wave sections and, consequently, the phase balance and impedance transformation accuracy. Parametric considerations indicate that a ±2% variation in line length corresponds to an electrical phase deviation of approximately $\pm 7^{\circ }$ at the unit-cell level. However, due to the symmetric architecture of the two-way and four-way Gysel structure, these deviations are partially self-compensating, resulting in a reduced net phase imbalance at the output ports. As a result, the overall phase variation in the four-way configuration remains within approximately $\pm 1^{\circ }$ around the center frequency, which is consistent with the simulated results presented in Fig 9. Similarly, dielectric constant variations introduce small shifts in electrical length and characteristic impedance. Sensitivity analysis shows that such variations have a secondary effect compared to geometric tolerances, leading to minor degradation in return loss and isolation (typically within 1–2 dB) without significantly impacting the overall combining efficiency or stability. These results indicate that the proposed impedance configuration and layout exhibit robust performance against realistic fabrication tolerances. This robustness is further enhanced by the use of moderate impedance values (avoiding excessively narrow high-impedance lines) and symmetric layout design, which together reduce sensitivity to manufacturing imperfections.

### 4.4. Summary

The simulation results confirm that the proposed four-way *Gysel* combiner meets the stringent requirements of L-band pulsed radar systems: low input reflection, high isolation, tight phase tracking, and good amplitude uniformity. The additional tolerance analysis further demonstrates that these performance metrics are maintained under realistic fabrication variations, confirming the robustness of the design. These characteristics validate the suitability of the design for kilowatt-class solid-state transmitter architectures.

## 5. Hardware implementation and thermal management

**Fig 11:**
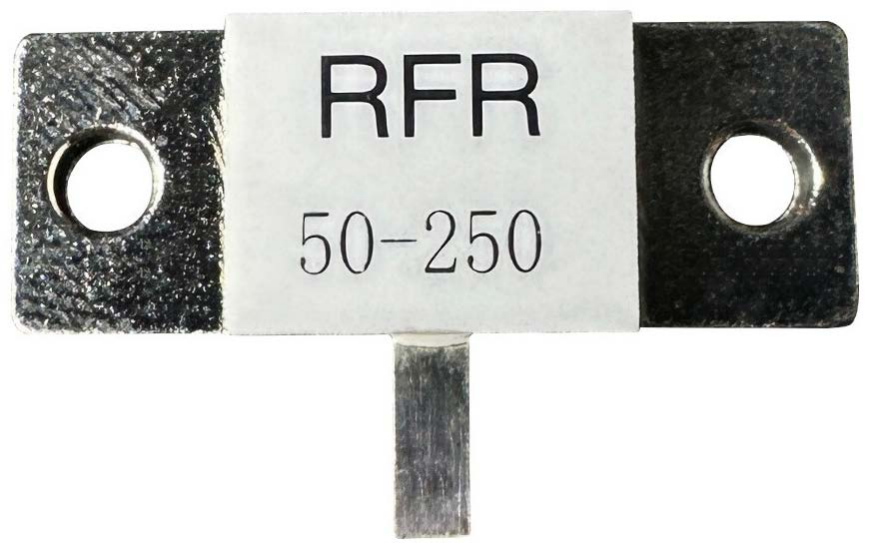
Flange-mounted RF termination resistor (50 Ω, 250 W) used as the isolation load in the *Gysel* network (photograph taken by the authors; commercial component).

Reliable kilowatt-class operation requires careful consideration of component selection, thermal interfaces, and mechanical integration. Fig 11 shows the high-power flange-mounted RF termination resistor used as the isolation load within the *Gysel* network. The metallic flange provides a direct heat-transfer path to the aluminum baseplate, minimizing thermal resistance and enhancing dissipation during imbalance or fault conditions. Its placement at the termination of the isolation branch ensures short RF paths while enabling efficient cooling. According to manufacturer specifications, the device supports broadband DC-GHz operation with a continuous 250 W dissipation rating [24]. For operation at L-band frequencies, the parasitic inductance of the flange-mounted resistor package is negligible relative to the 50 Ω system impedance, ensuring that its RF behavior closely approximates an ideal matched termination within the operating band.

**Fig 12:**
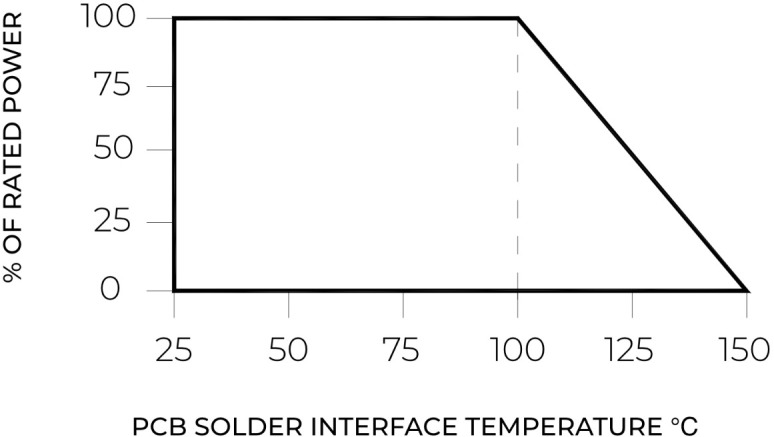
Power derating curve for the flange-mounted resistor versus PCB solder-interface temperature.

The resistor’s power derating curve, shown in Fig 12, indicates that the component maintains its full rated power up to approximately 100 °C. Beyond this temperature, the permissible power decreases linearly to zero at 150 °C. This behavior underscores the importance of maintaining a low thermal impedance path from the resistor to the baseplate and ensuring that the enclosure provides sufficient conduction cooling under pulsed high-power operation. Under the specified pulsed operating condition (10% duty cycle), the effective average power dissipated in the isolation resistors is significantly reduced, thereby maintaining the junction temperature within the safe operating region.

**Fig 13:**
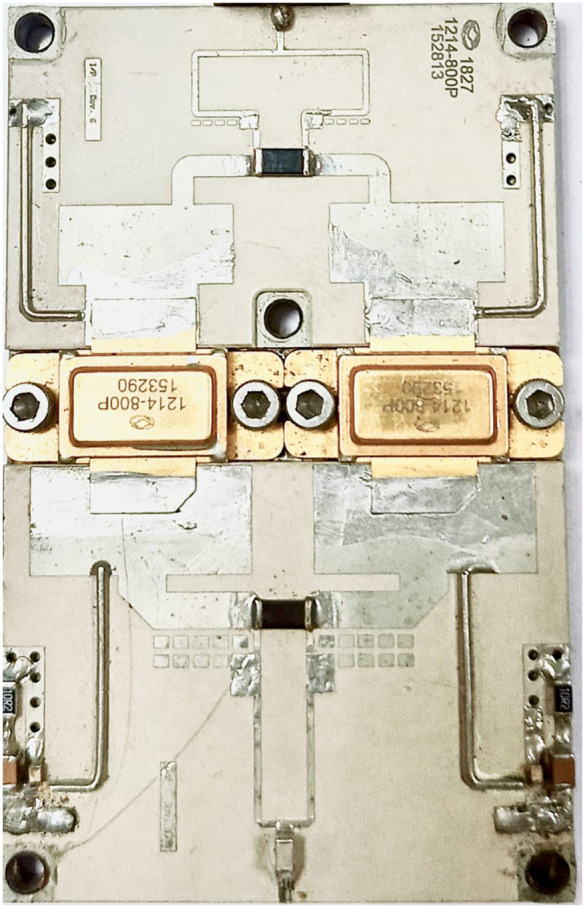
Power Amplifier Module (PAM) based on the Microsemi 1214-800P device. Each PAM delivers 800 W pulsed RF output in the L-band (photograph taken by the authors; commercial system).

Fig 13 depicts one of the four Power Amplifier Modules (PAMs), each based on the Microsemi 1214-800P device [25]. Each module supplies 800 W pulsed output over 1.2–1.4 GHz, using 300 µs pulses at a 10% duty cycle. The PAM assembly features short RF traces to reduce insertion loss, wide low-inductance DC feed lines to support high current transients, and distributed capacitor banks for immediate pulse energy delivery. The PAMs are mounted adjacent to the combiner ports to minimize interconnect inductance and maintain phase coherence. When integrated with the four-way *Gysel* combiner, the system achieves a peak output of 3 kW. The use of pulsed excitation further alleviates steady-state thermal loading, enabling reliable high peak-power operation without exceeding the thermal limits of the substrate and components.

### 5.1. Critical role of these components

The flange-mounted isolation resistor provides the primary mechanism for dissipating odd-mode energy, thereby ensuring high port-to-port isolation and thermal robustness during mismatch events. The derating characteristics emphasize the necessity of maintaining a controlled thermal environment to prevent resistor overheating. Meanwhile, the PAM integration strategy, short RF paths, wide DC buses, and local energy storage, enables efficient pulsed operation. Together, these elements form the foundation of a reliable high-power solid-state transmitter.

### 5.2. Thermal reliability considerations

Although direct long-term aging and lifetime measurements were not conducted, the design incorporates several features to enhance reliability under repeated pulsed high-power operation. These include: (i) direct heat sinking of isolation resistors through flange mounting, (ii) distributed ground-via arrays to improve thermal conduction into the ground plane, and (iii) operation under reduced average power conditions due to pulsed duty cycling. These design measures significantly mitigate thermal cycling stress and reduce the likelihood of material fatigue, solder joint degradation, and resistor performance drift over time. As a result, the proposed design is expected to maintain stable performance under practical radar operating conditions involving repeated pulsed excitation, within the limits of the component ratings and thermal design constraints.

## 6. Fabrication and hardware integration

This section describes the fabrication and integration processes of the proposed work.

### 6.1. Substrate, metallization, and stack-up

The combiner PCB is fabricated on RO4003C^TM^, which features a dielectric constant of $\varepsilon_{r}=3.38\pm 0.05$ and a low loss tangent (tanδ≈0.0027 at 10 GHz). These properties ensure stable electrical performance and manageable thermal behavior at L-band frequencies. The specified tolerance in dielectric constant is explicitly considered in the design phase, as it introduces small variations in electrical length and impedance that can affect phase balance and matching. The design uses 35 µm (1 oz) copper cladding with ENIG finish to achieve low contact resistance and high solderability. All transmission-line segments are dimensioned to provide λ/4 electrical lengths at 1.3 GHz, and transitions between PAM interfaces and microstrip tracks are width-matched to minimize discontinuities. Grounded isolation branches incorporate 50 Ω, 250 W flange-mounted resistors thermally bonded to the baseplate for enhanced heat extraction.

### 6.2. Mechanical assembly and thermal path

The PCB is mounted onto a precision-machined aluminum baseplate using countersunk screws and star washers at points aligned with dense ground-via regions. This ensures both low thermal resistance and reduced RF impedance to ground. The four PAMs are arranged in a linear configuration with minimal interconnect length to control insertion loss and phase offsets. Wide DC supply buses and distributed capacitor banks ensure stable high-current delivery during pulsed operation (300 µs, 10% duty cycle). Thermal interface material is applied beneath each PAM flange and isolation resistor to improve heat spreading. This assembly approach minimizes thermal gradients across the structure and reduces the impact of thermal cycling on solder joints and RF interconnects during repeated pulsed operation. The metal enclosure provides a low-inductance current return path and additional environmental shielding.

### 6.3. Final integration

**Fig 14:**
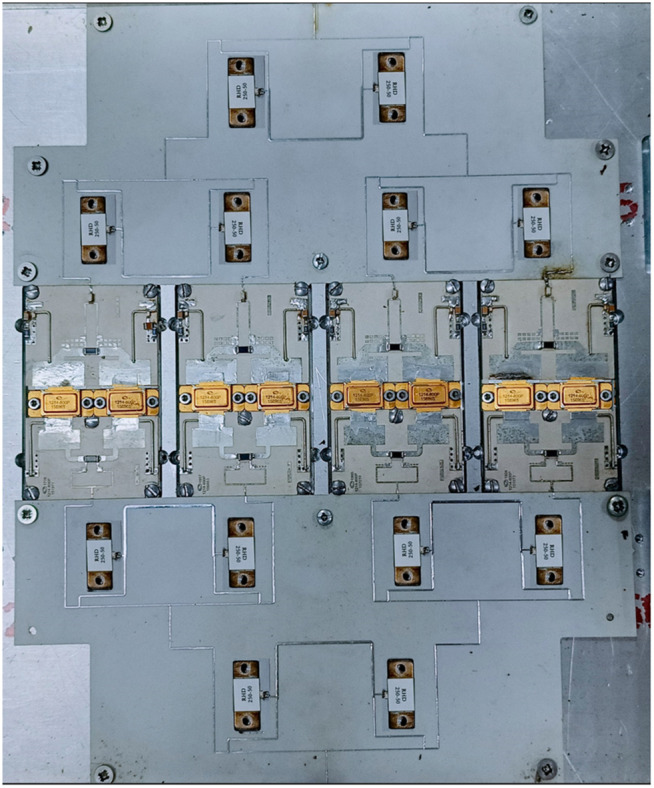
Final integration of the four PAMs with the CST-optimized four-way *Gysel* combiner on RO4003C^TM^ (The proposed fabricated system).

Fig 14 shows the final integrated assembly. Key implementation features include: (i) precise track-width matching between PAM output interfaces and combiner ports; (ii) grounded isolation branches equipped with flange-mounted power resistors; (iii) dense via carpets beneath high-current and high-thermal-load regions; and (iv) capacitor banks placed strategically to minimize supply droop during RF pulses. Careful layout symmetry and equalized path lengths are maintained during assembly to preserve phase coherence and reduce the accumulation of tolerance-induced imbalance across the cascaded structure.

### 6.4. Implemented impedances and layout constraints

The final impedance set— $Z_{1}=50\Omega ,\;Z_{2}=75\Omega ,\;Z_{3}=50\Omega ,\;Z_{4}=30\Omega ,\;R=50\Omega$—is obtained from CST optimization performed under the spatial and mechanical constraints imposed by PAM placement. Departing from canonical *Gysel* impedance ratios enhances mid-band isolation while maintaining acceptable insertion loss. The fixed spacing between PAM connectors and the limited real estate available for routing necessitated simulation-driven refinement. These constraints were incorporated into the optimization loop to ensure that the final design remains robust under practical assembly tolerances and mechanical misalignments.

### 6.5 Manufacturability notes

To reduce sensitivity to fabrication tolerances, narrow high-impedance microstrip sections were avoided, bends were mitered, and transitions were chamfered. Ground vias were stitched with a pitch of ≤1.5 mm in regions of high current density, consistent with the substrate manufacturer’s guidelines. All RF connectors and mechanical fasteners were installed with calibrated torque, and the completed assembly underwent inspection for solder voids, lifted pads, and enclosure clearance to ensure reliable operation. These manufacturability considerations further enhance reproducibility and minimize performance degradation due to fabrication-induced variations, supporting consistent operation across multiple fabricated units.

## 7. Measurement setup and procedures

This section shows the setup and the measurement procedures involved in this work.

1. *Step 1 (Instrumentation)*: S-parameters were measured using a calibrated Vector Network Analyzer (VNA) equipped with high-stability test cables and precision coaxial adapters. A dual directional coupler and high-power RF power meter were utilized for pulsed measurements. To ensure thermal stability during imbalance testing, coaxial terminations rated at ≥250 W per port were mounted directly to the baseplate. Oscilloscope triggering was synchronized with the pulse control circuitry to enable in-pulse monitoring of amplitude and phase. The pulsed RF excitation was generated using a pulsed frequency generator configured to produce 300 µs pulses at the specified duty cycle, ensuring consistent pulse operation across all PAM channels.
2. *Step 2 (Calibration and De-Embedding)*: A short-open-load-through (SOLT) calibration was performed at the coaxial reference planes using manufacturer-certified calibration standards. All connectors were torqued to specification to ensure repeatability. Fixture and adapter effects were removed via de-embedding using reference jig measurements, ensuring that the reported S-parameters correspond accurately to the *Gysel* combiner ports. Reference planes were defined at the PAM-combiner interfaces for splitter-mode tests and at the common port for combiner-mode characterization. The reference jig consisted of through and open structures with identical connector interfaces, allowing systematic removal of cable and adapter contributions. Residual de-embedding errors are estimated to be within ±0.2 dB in magnitude and $\pm 0.5^{\circ }$ in phase, consistent with the repeatability limits of the measurement setup.
3. *Step 3 (Low-Power Characterization)*: With all output ports terminated in 50 Ω loads, low-power validation focused on confirming the following specifications:
  - Input return loss $S_{11}\leq -15$ dB over 1.2–1.4 GHz.
  - Magnitude of $S_{2,1}-S_{5,1}$: target −6.64±0.3 dB at 1.3 GHz (ideal division: −6.02 dB).
  - Inter-port isolation $S_{ij}\geq -25$ dB at mid-band.
  - Insertion-phase spread within $\pm 1^{\circ }$ at 1.3 GHz.

Time gating and VNA phase referencing were used to suppress fixture reflections and ensure accurate amplitude and phase measurements.

4. *Step 4 (Pulsed High-Power Validation)*: For pulsed operation (300 µs pulses, 10% duty cycle), the following procedure was used:
  (a) Each PAM was driven with a calibrated RF input while gate bias and drain voltage stability were monitored.
  (b) Forward and reflected power were measured using the dual directional coupler at the common port.
  (c) An oscilloscope synchronized to the pulse trigger captured in-pulse amplitude and phase for each branch. Pulse droop was monitored over the 300 µs interval and found to be negligible, indicating sufficient DC supply stability and effective decoupling within the PAM assemblies.
  (d) Average power was extracted from peak power via:
    $$\begin{array}{c} P_{avg}=0.1\times P_{peak}, \end{array}$$ (3)
    reflecting the 10% duty cycle.
  (e) Isolation performance under imbalance was evaluated by detuning one PAM and monitoring the resulting odd-mode absorption at the isolation branches.

Safety interlocks and thermal shutdown mechanisms were engaged during all high-power tests. High-power terminations were baseplate-mounted to enhance heat dissipation.

The ideal combined output power from the four PAMs is 3.2 kW, assuming each module delivers 800 W. However, due to integration losses in the combiner structure, including conductor, dielectric, and residual mismatch losses, the measured output power is approximately 3 kW. This corresponds to an overall combining efficiency of about 93.75%, equivalent to an insertion loss of approximately 0.28 dB, which is consistent with expected performance in high-power planar microstrip combiners.

5. *Step 5 (Data Reduction and Comparison)*: Measured S-parameters were exported in Touchstone format and compared directly to CST simulation results. Small frequency shifts observed in the measurements were attributed to fabrication tolerances, substrate variations, and connector repeatability. The optimized impedance set ($Z_{2}=75\Omega ,\;Z_{4}=30\Omega$) exhibited the expected behavior: increased mid-band isolation with a modest reduction in operational bandwidth. Fine electrical-length trimming ensured phase alignment within $\pm 1^{\circ }$ at 1.3 GHz. The measured combining efficiency and insertion loss show good agreement with simulation trends, with minor deviations attributed to fabrication tolerances and connector losses.
6. *Step 6 (Uncertainty and Repeatability)*: Successive measurements demonstrated stability within ±0.2 dB in magnitude and $\pm 0.5^{\circ }$ in phase. Primary sources of uncertainty included cable flex, connector torque variations, incomplete de-embedding of fixture parasitics, and temperature drift during pulsed operation. These uncertainty levels are consistent with the estimated de-embedding accuracy and confirm the reliability of the reported measurement results.

### 7.1. Measurement setup

**Fig 15:**
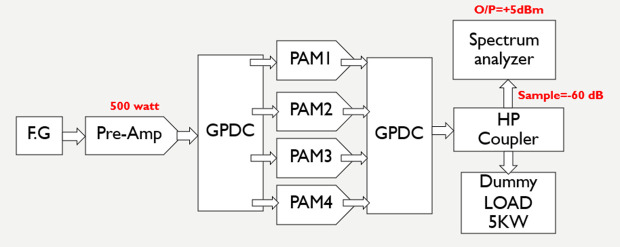
Block diagram of the high-power measurement setup for validating the four-way *Gysel* combiner integrated with four PAMs.

Fig 15 shows the high-power measurement configuration used to validate the CST-optimized four-way *Gysel* combiner integrated with the four PAMs. The setup consists of:

- *Signal Source:* A function generator followed by a pre-amplifier delivering 500 W of drive power to the GPDC input.
- *PAM Array:* Four PAMs connected to the GPDC splitter, each delivering 800 W pulsed RF output.
- *Combining Stage:* A second GPDC module combines the four PAM outputs to achieve a peak signal at the common port. Ideally, the total combined output power is expected to be 3.2 kW, corresponding to four PAMs each delivering 800 W. However, due to practical combining losses, the measured output power is approximately 3 kW, which corresponds to a combining efficiency of about 93.75%.
- *Load and Sampling Chain:* The combined output is terminated in a 5 kW dummy load, with a high-power coupler extracting a −60 dB sampled signal for monitoring.
- *Instrumentation:* A spectrum analyzer used to measure output power, pulse characteristics, and spectral purity. Time-domain measurements using a synchronized oscilloscope were employed to verify pulse width, amplitude stability, and transient behavior.

### 7.2. Measurement and validation

S-parameters were measured using SOLT-calibrated VNA instrumentation. The two-way prototype confirmed equal-power division and high isolation, while the full four-way network demonstrated stable amplitude and phase behavior after de-embedding. Measured results closely follow CST predictions and the expected theoretical characteristics of *Gysel* dividers [1,2].

#### 7.2.1. Low-power VNA validation.

**Fig 16:**
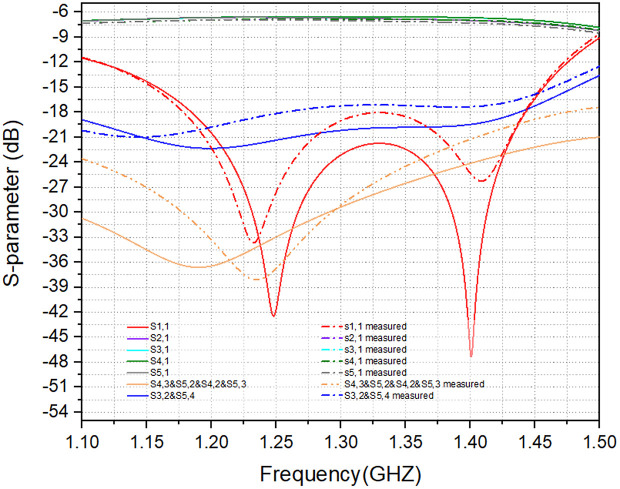
Comparison of simulated (solid) and measured (dashed) S-parameters for the four-way *Gysel* combiner under low-power VNA testing.

Fig 16 compares CST-simulated and measured S-parameters of the four-way combiner over 1.1–1.5 GHz. Solid lines correspond to CST data, while dashed lines represent measurements. The validation shows:

- *S*_11_ better than −15 dB across the operating band,
- insertion loss within 0.6 dB of the ideal −6.02 dB split,
- inter-port isolation greater than −25 dB, consistent with simulation.

Minor discrepancies are attributed to fabrication tolerances and connector repeatability.

#### 7.2.2 Pulsed operation.

Under pulsed operation (300 µs pulses at 10% duty cycle), the average transmitted RF power was 300 W from a 3 kW peak, effectively reducing thermal stress. The pulsed RF excitation was generated using a pulsed frequency generator configured to produce stable 300 µs pulses at the specified duty cycle across all PAM channels. Triggered detection captured the in-pulse phase and amplitude response, and minor electrical-length adjustments were made to maintain phase tracking within $\pm 1^{\circ }$ at 1.3 GHz, consistent with high-power microstrip design guidelines [13]. The stable RF output during the pulse interval indicates minimal amplitude variation, confirming adequate DC supply regulation and effective energy storage within the PAM modules.

### 7.3. Power handling and thermal budget

Grounded isolation branches play a critical role in absorbing odd-mode energy and localizing dissipation, thereby enhancing fault tolerance under imbalance conditions. The 50 Ω, 250 W flange-mounted resistors bonded directly to the baseplate provide substantial thermal headroom during mismatch events, with datasheet specifications confirming broadband DC-GHz operation at their rated power. Dense via carpets beneath high-current regions further reduce thermal resistance by spreading heat into the ground plane and underlying metal baseplate. Considering the measured combining efficiency of approximately 93.75%, the associated power dissipation (approximately 200 W) is distributed across conductor losses, dielectric losses, and isolation elements, remaining within the safe thermal operating limits of the structure. These design measures align with the classical high-power rationale of the *Gysel* topology [1], while also supporting modern improvements in Average Power Handling Capability (APHC) for planar RF structures [13].

### 7.4. System integration and discussion

Employing the *Gysel* architecture as both splitter and combiner enables staged absorption of odd-mode power throughout the signal path, offering enhanced stability compared to Wilkinson-type dividers that rely on floating resistors. The measured results confirm that the proposed planar implementation maintains stable amplitude and phase characteristics under both low-power and high-power pulsed operation, with performance deviations consistent with expected fabrication and measurement tolerances. Practical implementation trade-offs include relaxing transmission-line impedances toward 90 Ω to reduce conductor loss and manufacturing sensitivity, or introducing multi-section transformers to improve matching bandwidth [2,20]. For applications requiring extremely high power density, ridge-waveguide and quasi-planar coaxial *Gysel* structures present alternative solutions with superior thermal margins and lower conductor loss [8,9]. Nevertheless, the planar RO4003C implementation developed here strikes an effective balance between manufacturability, thermal performance, and coherent L-band combining. These results validate the proposed design as a practical solution for compact solid-state radar transmitters requiring reliable kilowatt-class performance.

## 8. Conclusion

This work presented the design, optimization, fabrication, and experimental validation of a planar four-way *Gysel* divider/combiner for L-band pulsed radar transmitters. Implemented on an RO4003C substrate, the proposed architecture achieves a peak output power of 3 kW while maintaining low insertion loss, high inter-port isolation, and tight phase coherence—key requirements for coherent combining in modern solid-state transmitter arrays. The measured combining efficiency of approximately 93.75% (corresponding to an insertion loss of about 0.28 dB) confirms the effectiveness of the proposed design for high-power planar implementations. Grounded isolation branches, flange-mounted high-power resistors, and thermally optimized via structures enable stable kilowatt-class operation under pulsed and mismatch conditions. Full-wave electromagnetic simulations show strong agreement with measured S-parameters and in-pulse behavior, demonstrating that the power-aware impedance optimization and thermal design methodology adopted in this work yields robust and repeatable high-power performance. Additional tolerance analysis indicates that the design maintains its performance under realistic fabrication variations, with phase imbalance remaining within acceptable limits due to the symmetric architecture and moderate impedance values. Overall, the results confirm that the *Gysel* topology, when carefully optimized and thermally engineered, provides an efficient, compact, and manufacturable planar solution for next-generation kilowatt-class L-band radar transmitters. Unlike conventional waveguide-based solutions, the proposed approach offers a favorable balance between performance, size, and implementation complexity, making it particularly suitable for compact solid-state systems. Although the proposed design achieves demonstrated kilowatt-class performance, several avenues remain for further enhancement. These include the incorporation of multi-section impedance transformers to extend the useful bandwidth, the integration of planar thermal spreaders or metal-backed vias to further increase the average power handling capability, and the implementation using GaN-based power amplifier modules to enable higher peak power, improved efficiency, and enhanced thermal headroom. Furthermore, the modular architecture provides a pathway toward scaling to higher port counts (e.g., 8-way or 16-way configurations), although this introduces additional challenges in phase synchronization and thermal distribution that warrant further investigation. Such improvements are expected to provide additional design margin and broaden the applicability of the proposed approach in future high-power solid-state radar systems.

## Supporting information

S1 Dataset RF power divider/combiner measurement and simulation dataset. Compressed archive containing the underlying verification data for the proposed planar four-way *Gysel* power networks. The dataset specifically includes the text document Insertion Phase (4-Way) containing transmission phase data for the four-way combiner, alongside the Microsoft Excel spreadsheet 4_Way Measured and Simulated S-parameter Results which provides the comprehensive comparative measured versus simulated S-parameter datasets for the complete four-way system. Inter-port isolation and matching characteristics of the constituent stages are detailed in the text documents S32-2-way, which maps the isolation (*S*_32_) of the two-way Gysel cell, and 2-way-S11-Matchig, which tracks the input return loss (*S*_11_) and impedance matching performance of the two-way prototype. Furthermore, the archive contains standalone verification text files including Insertion Phase (2-Way) for the transmission phase data of the individual cell and Insertion Loss outlining amplitude attenuation and transmission efficiency characteristics. Finally, the Touchstone file 1214-800P_synthetic_GHz.s2p provides the complete multi-port scattering parameters across the targeted L-band frequency sweep. Collectively, these files facilitate the independent reproduction, analysis, and validation of the reported RF performance using standard analytical software such as MATLAB, CST Studio Suite, Keysight ADS, or Microsoft Excel.
(RAR)

S2 File S2 Mechanical design of the proposed planar four-way *Gysel* power divider/combiner with integrated power amplifier modules. Three-dimensional model of the complete 4-way high-power Gysel combiner integrated inside the metallic enclosure. The housing provides mechanical support, improved thermal management, and high-power RF operation capability. It also shows the signal-routing and interconnection layout of the proposed combiner showing the transmission-line sections, isolation branches, and port locations used for power combining from four amplifier modules into a single output port.
(PDF)

S3 File S3 Assembly model of the proposed planar four-way *Gysel* power divider/combiner. Layout of the proposed four-way Gysel power divider/combiner realized on Rogers RO4003C substrate. The circuit comprises four equal power-division branches and isolation networks designed for high-power operation over the 1.1–1.5 GHz frequency range.
(PDF)
