# Supplementary figures and images for: Kilowatt-class planar four-way gysel combiner with power-aware impedance optimization for L-band pulsed radar
Source: PLoS One. 2026 Jul 24;21(7):e0354086. doi: 10.1371/journal.pone.0354086 (PMC13399331; doi:10.1371/journal.pone.0354086)

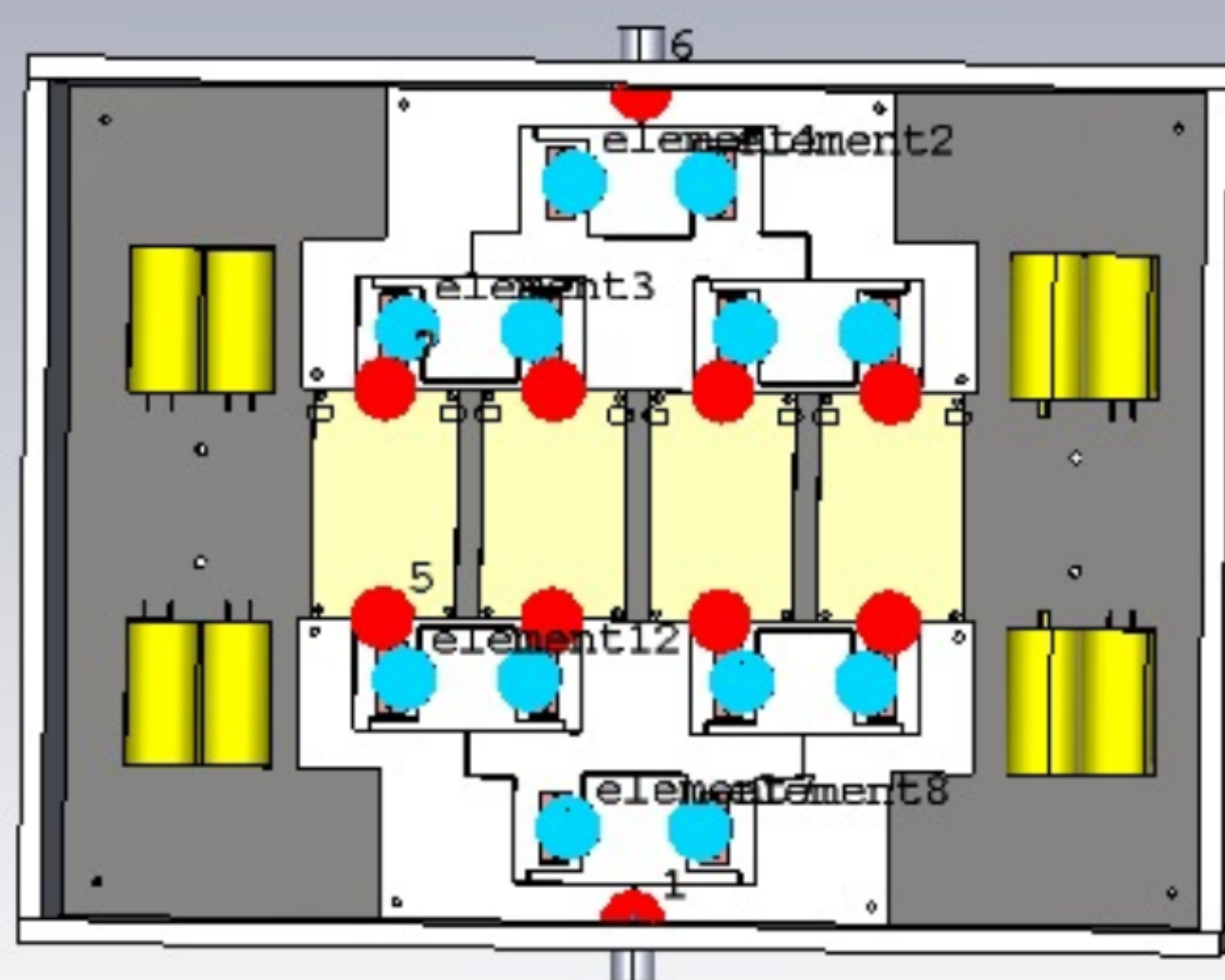

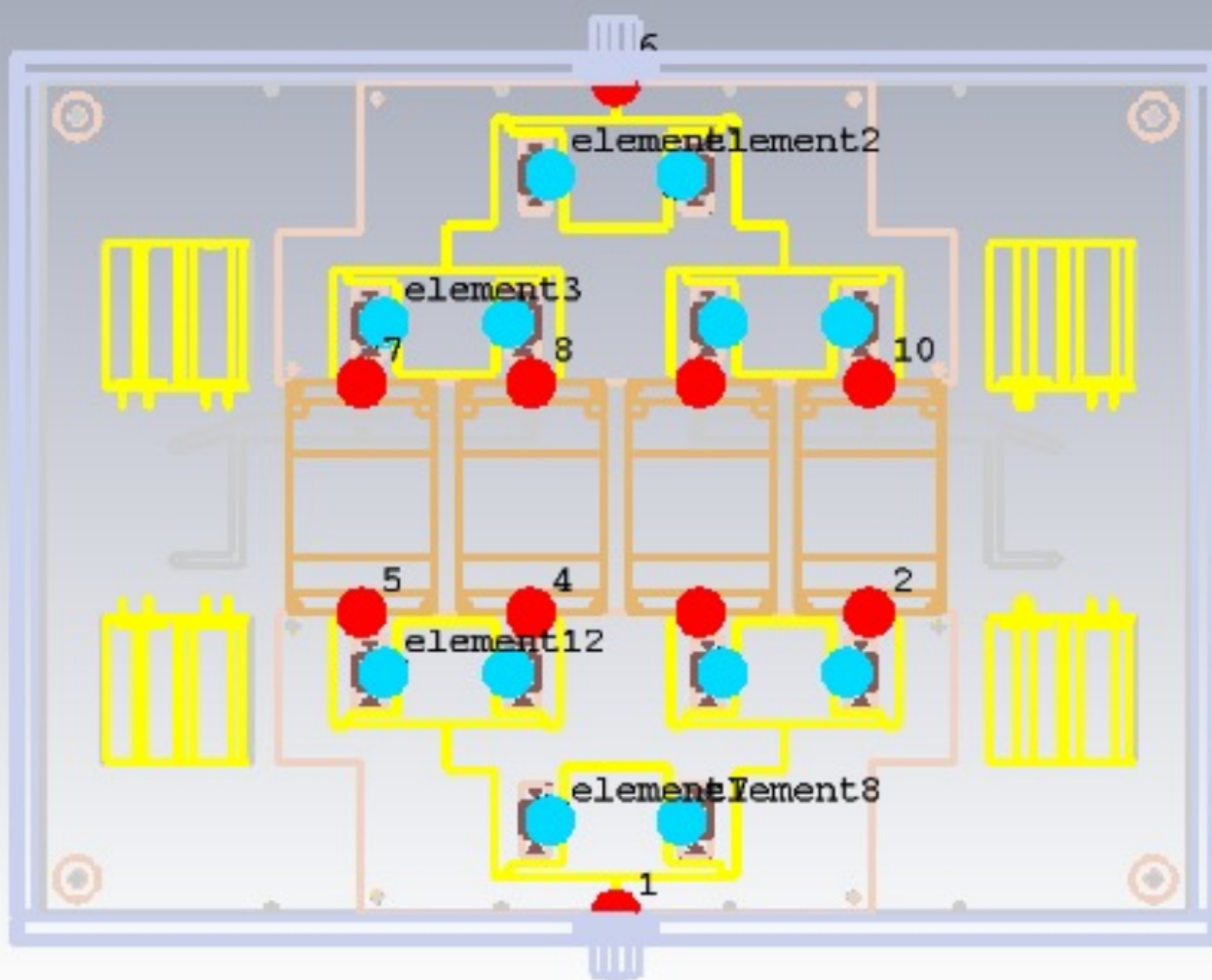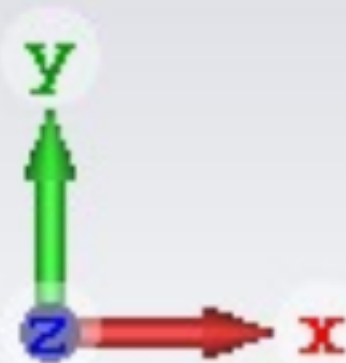

Supplement: S2 File — Three-dimensional model of the complete 4-way high-power Gysel combiner integrated inside the metallic enclosure. The housing provides mechanical support, improved thermal management, and high-power RF operation capability. It also shows the signal-routing and interconnection layout of the proposed combiner showing the transmission-line sections, isolation branches, and port locations used for power combining from four amplifier modules into a single output port. (PDF) [file pone.0354086.s002.pdf]

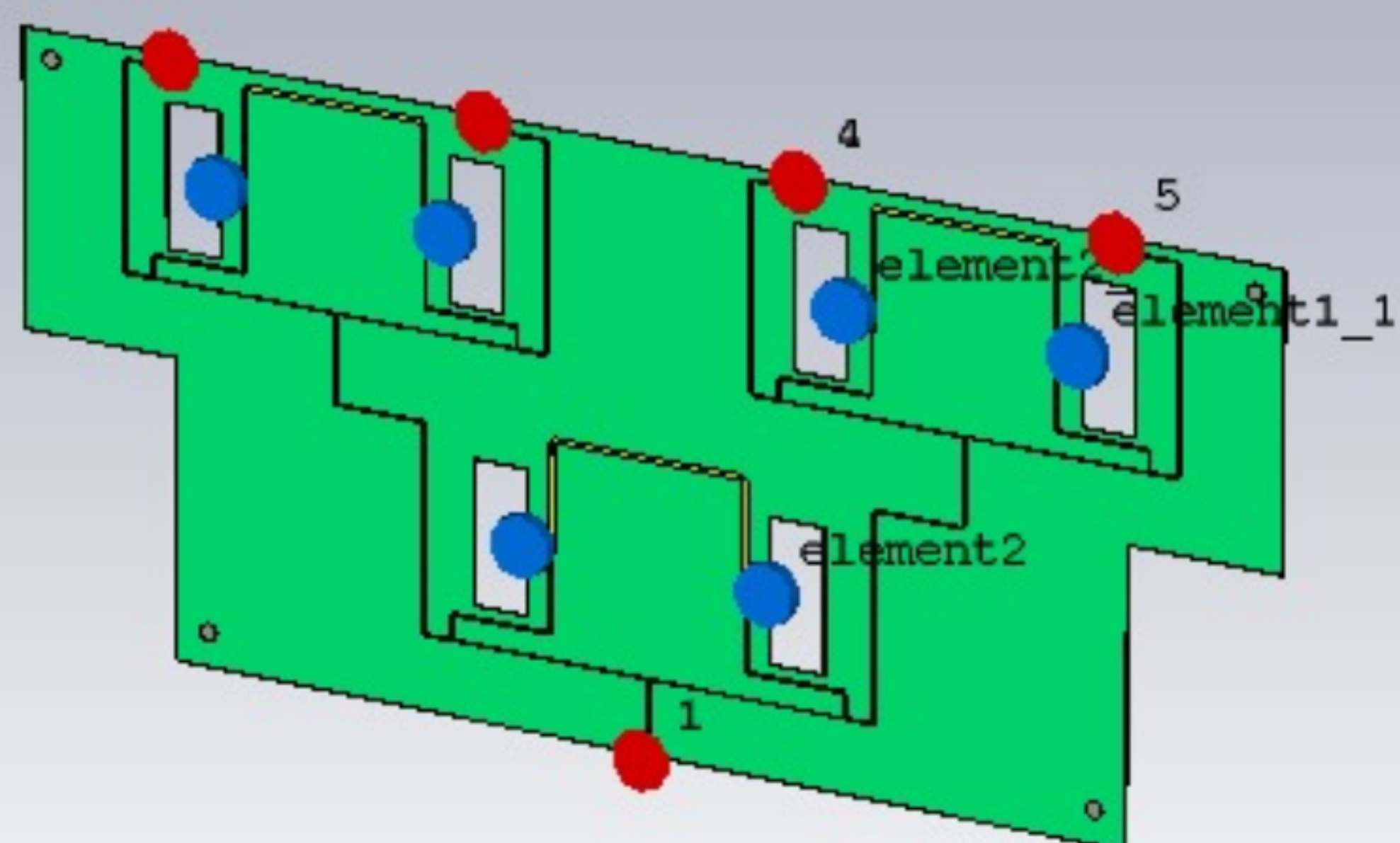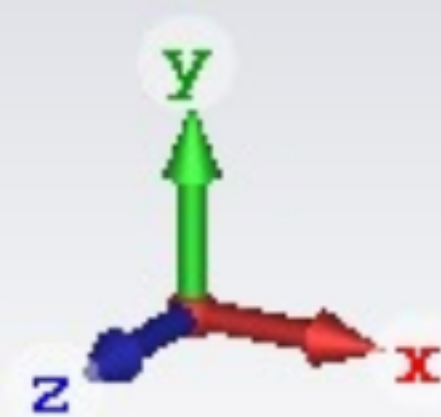

Supplement: S3 File — Layout of the proposed four-way Gysel power divider/combiner realized on Rogers RO4003C substrate. The circuit comprises four equal power-division branches and isolation networks designed for high-power operation over the 1.1–1.5 GHz frequency range. (PDF) [file pone.0354086.s003.pdf]
