# Supplementary material for: Kilowatt-class planar four-way gysel combiner with power-aware impedance optimization for L-band pulsed radar
Source: PLoS One. 2026 Jul 24;21(7):e0354086. doi: 10.1371/journal.pone.0354086 (PMC13399331; doi:10.1371/journal.pone.0354086)
Supplement: Attachment — Submitted filename: Response_Letter.pdf [file pone.0354086.s005.pdf]

Journal of Computers and Electrical Engineering
Manuscript ID: PONE-D-26-18991
Title: Kilowatt-Class Planar Four-Way Gysel Combiner with Power-Aware Impedance Optimization for L-Band Pulsed Radar

Response to Editor and Reviewers

We would like to sincerely thank the Editorial Office of **PLOS ONE** and the reviewers for their careful evaluation of our manuscript and for their constructive and insightful comments. These valuable suggestions have significantly contributed to improving the clarity, technical rigor, and overall quality of the work.

All comments have been carefully considered and thoroughly addressed in the revised manuscript. For ease of review, reviewer comments are shown in **black**, our responses are provided in blue, and all corresponding modifications in the manuscript are highlighted in red.

Response to the Editorial Office

1) **Comment: Please revise the manuscript based on the reviewers' comments.**

   We sincerely appreciate this guidance. In response, the manuscript has been comprehensively revised to address all reviewer comments in a systematic and thorough manner. Specifically, the revised manuscript includes: (i) a quantitative comparison positioning the proposed design with respect to recent studies, (ii) a detailed tolerance analysis evaluating robustness under fabrication variations, (iii) improved clarification of the measurement setup and pulsed operation, and (iv) a more explicit discussion of high-power performance, including combining efficiency and loss mechanisms. Collectively, these revisions significantly enhance both the technical depth and clarity of the manuscript.

Response to Editorial Office Requirements

1) **Formatting requirements**

   We confirm that the manuscript has been carefully reviewed and updated to fully comply with PLOS ONE formatting requirements, including structure, figure presentation, and file naming conventions.

2) **Code sharing**

We confirm that no custom code underpins this work. The study is based on CST electromagnetic simulations and experimental measurements; therefore, no code sharing is required.

3) **Data availability**

All relevant data required to reproduce the results are included within the manuscript and Supporting Information files, including design parameters, simulation results, and experimental measurements.

4) **Minimal dataset**

The submission includes the minimal dataset necessary to replicate the reported findings, including numerical values underlying figures and performance metrics.

5) **Copyright of figures**

All figures have been reviewed and confirmed to be original. They were simulated, measured, or generated by the authors. Figure captions have been updated accordingly to ensure full compliance with CC BY 4.0 requirements.

6) **Suggested citations**

Relevant suggested references have been incorporated where appropriate to strengthen the literature review.

Response to Reviewer 1

1) **Comment: The novelty of the work is not clearly justified.**

We thank the reviewer for highlighting this important point. We agree that the novelty statement should be carefully framed to avoid overstatement. Accordingly, the manuscript has been revised to present a more accurate and defensible positioning of the contribution within the existing literature.

Revision in manuscript: The statement has been updated to: "the proposed design represents a rare example of a planar microstrip Gysel combiner experimentally validated for reliable kilowatt-class operation."

2) **Comment: Please consider recent PCB-integrated approaches and cite the suggested work by Khan et al.**

   We thank the reviewer for this valuable suggestion and for highlighting the relevance of recent PCB-integrated approaches in high-reliability aerospace applications. We agree that such works provide important insights into compact planar implementation and thermal management challenges, which are directly relevant to the proposed kilowatt-class design. In response, the suggested work by Khan *et al.* has been incorporated into the literature review. This study is discussed to emphasize advanced PCB integration techniques and their role in enabling compact, thermally efficient, and highly integrated planar systems. The inclusion of this reference strengthens the contextual foundation of our work and better positions the proposed design within the broader landscape of modern PCB-based electromagnetic systems.

   Revision in manuscript: The following text has been added to Section 1.2: "Recent advances in PCB-integrated aerospace systems further highlight the importance of compact planar implementation and effective thermal management. For instance, Khan *et al.* demonstrated reconfigurable embedded planar structures for nanosatellite applications, where integration density and thermal constraints are critical design considerations. Such approaches underscore the relevance of advanced PCB-based integration techniques in achieving high-performance and reliable operation in compact RF systems."

3) **Comment: The manuscript lacks a quantitative comparison with related work.**

   We fully agree with the reviewer. To address this concern, we have introduced a quantitative comparison that includes representative planar, coaxial, and waveguide-based *Gysel* combiners. This comparison presents key performance metrics such as operating frequency, power handling capability, isolation, phase imbalance, and insertion loss, thereby enabling a clear and objective positioning of the proposed design within the current state-of-the-art.

   Revision in manuscript: A new quantitative comparison table (Table 1) has been added in the Literature Review section. The table includes representative works and summarizes key performance metrics, including frequency, output power capability (in equivalent CW values), isolation, phase imbalance, and insertion loss.

   In addition, the following discussion has been incorporated into the manuscript: "As shown in Table 1, waveguide and coaxial implementations typically achieve superior power handling capability and lower insertion loss due to their enhanced thermal mar-

Table 1: **Comparison with recent Gysel-based high-power dividers/combiners.**

**Ref**	**Year**	**Freq (GHz)**	**Topology**	**Power (W, CW)**	**ISO (dB)**	**PI**	**IL**	**Remarks**
[5]	2013	4–8	Planar Gysel	∼50–70	∼20	±1	∼1–1.5	2-Way wideband planar
[8]	2019	3–5	Ridge-waveguide Gysel	∼5000	15–25	±1–2	∼0.1–0.2	2-Way high-power, low-loss
[9]	2015	4–5.5	Rectangular coaxial waveguide	1000–2000	5–15	±5	∼0.7	4-way chained quasi-planar
[11]	2025	4.5–5.5	Filtering Gysel	∼50	20–30	±1	∼0.2	Integrated filtering functionality
[13]	2022	2.3–2.5	Planar APHC Gysel	∼100	20–25	±3–5	∼0.1–0.2	2-Way thermal-aware design
[23]	2025	0.38	Modified Gysel	∼500	∼23	±1–3	∼0.16	2-Way compact high-power
**This work**	2026	1.1–1.5	Planar RO4003C	∼300	>25	±1	∼0.4	4-Way , 3 kW peak

gins and reduced conductor losses. However, these approaches are generally associated with larger physical size and higher fabrication complexity. In contrast, planar implementations provide advantages in compactness, manufacturability, and system-level integration. The proposed design achieves a balanced trade-off by enabling kilowatt-class operation within a compact planar microstrip architecture while maintaining competitive isolation, phase coherence, and acceptable insertion loss."

4) **Comment: The tolerance sensitivity of the design is not sufficiently addressed.**

We appreciate this valuable suggestion. To address this concern, a dedicated tolerance analysis has been included to quantitatively evaluate the impact of fabrication variations on system performance.

Revision in manuscript: A new subsection titled "Tolerance Analysis" has been added. This section examines the effects of ±2% variations in transmission-line dimensions and

dielectric constant deviations. The results demonstrate that, due to the symmetric architecture, phase imbalance remains within approximately $\pm 1^\circ$, with only minor degradation observed in matching and isolation performance.

5) **Comment: The comparison with waveguide and coaxial Gysel implementations remains largely qualitative and lacks quantitative support.**

   We thank the reviewer for this constructive suggestion. We agree that a quantitative comparison strengthens the justification of the proposed planar implementation. Accordingly, the manuscript has been revised to include a detailed comparison with representative waveguide, coaxial, and planar Gysel-based combiners. This comparison highlights key performance metrics, including power handling capability, isolation, and implementation characteristics, which provide a clearer perspective on the trade-offs between different technologies.

   Revision in manuscript: A new comparison (Table 1 has been added in Section 1.2. The table demonstrates that while waveguide and coaxial implementations offer superior power handling and thermal margins, they incur significantly higher size, weight, and fabrication complexity. In contrast, the proposed planar design achieves a favorable balance between compactness, cost-effectiveness, and high-power performance.

6) **Comment: The phase imbalance specification of $\pm 1^\circ$ is not supported by tolerance analysis.**

   We appreciate this important observation. A detailed tolerance analysis has been included to evaluate the sensitivity of the design to fabrication variations and material uncertainties, ensuring reproducibility of the reported performance.

   Revision in manuscript: A new "Tolerance Analysis" subsection has been added (Section 4.3), where the effects of $\pm 2\%$ variations in transmission-line lengths and dielectric constant deviations are analyzed. The results indicate that the symmetric architecture effectively mitigates these perturbations, maintaining phase imbalance within approximately $\pm 1^\circ$. Specifically, line length variations of $\pm 2\%$ were found to introduce only minor phase deviations due to the equal electrical path design, while dielectric constant variations result in slight impedance mismatches that do not significantly degrade isolation or return loss. These results confirm the robustness of the proposed design under practical fabrication tolerances.

7) **Comment: The concept of "power-aware optimization" is unclear.**

   We thank the reviewer for this important comment. The manuscript has been revised

to clarify the distinction between conventional electromagnetic optimization and the proposed “power-aware” approach.

Revision in manuscript: The design methodology section now explicitly explains that the optimization incorporates additional constraints relevant to high-power operation, including current density distribution, thermal dissipation mechanisms, and sensitivity to fabrication tolerances. This extends beyond standard S-parameter optimization to ensure reliable performance under kilowatt-class power conditions.

8) **Comment: The de-embedding process is not sufficiently detailed; reference jig structure and residual errors should be clarified.**

We thank the reviewer for this important observation. We agree that providing additional details on the de-embedding procedure improves the transparency and credibility of the measurement results. Accordingly, the manuscript has been revised to clarify both the reference jig configuration and the associated residual uncertainties.

Revision in manuscript: Additional details have been included in the measurement section (Section 5.1). It is now stated that the de-embedding procedure was performed using a calibrated reference jig consisting of identical connector-launch transitions and transmission-line segments fabricated on the same RO4003C substrate as the device under test (DUT). The reference measurements were used to characterize and remove the parasitic effects of connectors, adapters, and feeding structures from the measured S-parameters. The residual uncertainty after de-embedding has been estimated to be within ± 0.2 dB in magnitude and $\pm 0.5^\circ$ in phase. These values are consistent with typical RF measurement tolerances at L-band frequencies and confirm that the results presented in Fig. 15 accurately represent the intrinsic performance of the proposed high-power Gysel combiner.

9) **Comment: Please discuss scalability.**

We thank the reviewer for this insightful suggestion. The manuscript has been updated to include a more comprehensive discussion on scalability, addressing both the feasibility and practical limitations of extending the proposed topology to higher port counts.

Revision in manuscript: It is now stated that the proposed architecture can be extended to higher port counts (e.g., 8-way or 16-way) using a hierarchical cascading approach. However, when employing the same dielectric substrate (RO4003C) and identical PAM modules, the resulting increase in total combined power may exceed the thermal and conductor loss handling capabilities of the planar structure. To address this limitation,

two practical solutions are proposed: (i) reducing the output power per PAM module to keep the total power within safe operating limits, and (ii) utilizing high-thermal-conductivity substrates (e.g., ceramic-based materials or metal-backed structures) to enhance power-handling capability. These considerations provide a realistic framework for scaling the design while maintaining reliable high-power operation.

Response to Reviewer 2

1) **Comment: The 200 W discrepancy (6.2% loss) is not explicitly accounted for.**

   We thank the reviewer for this important observation. We agree that the difference between the input and output power should be explicitly stated and clarified.

   Revision in manuscript: The manuscript has been revised to clearly explain that the total input power of 3.2 kW (from four PAMs each delivering 800 W) results in a measured output power of approximately 3 kW. This difference corresponds to an overall combining loss. The combining efficiency is now explicitly stated as 93.75%, which corresponds to an insertion loss of approximately 0.28 dB. This value is consistent with expected conductor, dielectric, and residual mismatch losses in high-power planar microstrip combiners.

2) **Comment: No thermal measurements are presented.**

   We appreciate this observation. While direct thermal measurements were not conducted, the manuscript has been updated to clarify the thermal management approach and justify the expected thermal performance.

   Revision in manuscript: It is now clarified that thermal performance is ensured through design features including flange-mounted high-power resistors with direct heat sinking, dense ground-via arrays, and operation under pulsed conditions (10% duty cycle), which significantly reduces average power dissipation. These implementation strategies provide effective thermal management within the operating range of the system.

3) **Comment: Were the flange-mounted resistors characterized for inductance at L-band frequencies?**

   We thank the reviewer for raising this important point. The RF behavior of the flange-mounted resistor has now been clarified in the manuscript.

   Revision in manuscript: A statement has been added indicating that the parasitic inductance of the flange-mounted resistor is negligible at L-band frequencies relative

to the 50 Ω system impedance. Therefore, the resistor behaves effectively as an ideal termination within the operating frequency range.

4) **Comment: What is the combining efficiency at 3 kW peak?**

   We appreciate this comment. The combining efficiency has now been explicitly stated in the revised manuscript.

   Revision in manuscript: The combining efficiency is reported as approximately 93.75%, calculated from a total input power of 3.2 kW and an output power of 3 kW, corresponding to an insertion loss of approximately 0.28 dB.

5) **Comment: How was the 300 μs pulse verified? Was pulse droop characterized?**

   We thank the reviewer for this insightful question. The manuscript has been revised to clarify the pulse generation and stability assessment methodology.

   Revision in manuscript: It is now stated that the pulsed RF excitation was generated using a pulsed frequency generator configured to produce 300 µs pulses at a 10% duty cycle. The stability of the RF output during the pulse interval was observed to be consistent, with no noticeable amplitude variation, indicating negligible pulse droop and sufficient DC supply regulation within the PAM modules.

Conclusion

We believe that all comments from the Editor and reviewers have been carefully considered and fully addressed. The manuscript has been substantially improved in terms of clarity, technical rigor, and completeness. In particular, we have strengthened the literature review, introduced a quantitative comparison with state-of-the-art designs, clarified the measurement methodology, and provided additional analysis on tolerance sensitivity, scalability, and high-power performance. We sincerely appreciate the time and effort invested by the Editor and reviewers in evaluating our work. Their insightful feedback has been invaluable in enhancing the quality of this manuscript. We respectfully submit the revised version for reconsideration and hope it is now suitable for publication in **PLOS ONE**.

All authors have read and approved the changes made to the manuscript. We hope that the revised paper is now suitable for inclusion in the Journal of Computers and Electrical Engineering and we look forward to hearing from you.

Sincerely,

On behalf of all authors,

Syed Tariq Shah
School of Computer Science & Electronic Engineering
University of Essex, Colchester, United Kingdom
Email: syed.tariq@essex.ac.uk

Date: June 14, 2026
