# Supplementary material for: Kilowatt-class planar four-way gysel combiner with power-aware impedance optimization for L-band pulsed radar
Source: PLoS One. 2026 Jul 24;21(7):e0354086. doi: 10.1371/journal.pone.0354086 (PMC13399331; doi:10.1371/journal.pone.0354086)
Supplement: Peer review history

# Peer review: Decision Letter 0

**DOI:** 10.1371/journal.pone.0354086.r001

**Authors:** Muhammad Salman

5 Jun 2026

PONE-D-26-18991

Kilowatt-Class Planar Four-Way Gysel Combiner with Power-Aware Impedance Optimization for L-Band Pulsed Radar

PLOS One

Dear Dr. Shah,

Thank you for submitting your manuscript to PLOS ONE. After careful consideration, we feel that it has merit but does not fully meet PLOS ONE’s publication criteria as it currently stands. Therefore, we invite you to submit a revised version of the manuscript that addresses the points raised during the review process.

==============================

**ACADEMIC EDITOR:**

After reviewing your manuscript along with the two reviewer reports, I believe that while the work has technical merit, it needs major revisions to meet PLOS ONE's publication standards.

Please address the technical clarifications and concerns raised by the reviewers. Specifically, the need for a quantitative comparison with waveguide combiners, a tolerance analysis of potential fabrication errors, an explanation of the 200W power discrepancy, and a discussion of long-term reliability and thermal cycling for pulsed operation. Also, tolerance/fabrication analysis, and thermal reliability discussion.

Finally, the authors should update the introduction and literature review to include recent works from 2023 to 2026, as the current references are limited. Also, strengthen the quantitative comparison with state-of-the-art planar and waveguide combiners.

==============================

Please submit your revised manuscript by Jul 20 2026 11:59PM. If you will need more time than this to complete your revisions, please reply to this message or contact the journal office at plosone@plos.org. When you're ready to submit your revision, log on to https://www.editorialmanager.com/pone/ and select the 'Submissions Needing Revision' folder to locate your manuscript file.

Please include the following items when submitting your revised manuscript:

- A letter that responds to each point raised by the academic editor and reviewer(s). You should upload this letter as a separate file labeled 'Response to Reviewers'.
- A marked-up copy of your manuscript that highlights changes made to the original version. You should upload this as a separate file labeled 'Revised Manuscript with Track Changes'.
- An unmarked version of your revised paper without tracked changes. You should upload this as a separate file labeled 'Manuscript'.

If you would like to make changes to your financial disclosure, please include your updated statement in your cover letter. Guidelines for resubmitting your figure files are available below the reviewer comments at the end of this letter.

If applicable, we recommend that you deposit your laboratory protocols in protocols.io to enhance the reproducibility of your results. Protocols.io assigns your protocol its own identifier (DOI) so that it can be cited independently in the future. For instructions see: https://journals.plos.org/plosone/s/submission-guidelines#loc-laboratory-protocols. Additionally, PLOS ONE offers an option for publishing peer-reviewed Lab Protocol articles, which describe protocols hosted on protocols.io. Read more information on sharing protocols at https://plos.org/protocols?utm_medium=editorial-email&utm_source=authorletters&utm_campaign=protocols.

As the corresponding author, your ORCID iD is verified in the submission system and will appear in the published article. PLOS supports the use of ORCID, and we encourage all coauthors to register for an ORCID iD and use it as well. Please encourage your coauthors to verify their ORCID iD within the submission system before final acceptance, as unverified ORCID iDs will not appear in the published article. *Only*  the individual author can complete the verification step; PLOS staff *cannot*  verify ORCID iDs on behalf of authors.

We look forward to receiving your revised manuscript.

Kind regards,

Muhammad Salman

Guest Editor

PLOS One

Journal Requirements:

When submitting your revision, we need you to address these additional requirements.

1. Please ensure that your manuscript meets PLOS ONE's style requirements, including those for file naming. The PLOS ONE style templates can be found at

https://journals.plos.org/plosone/s/file?id=wjVg/PLOSOne_formatting_sample_main_body.pdf and https://journals.plos.org/plosone/s/file?id=ba62/PLOSOne_formatting_sample_title_authors_affiliations.pdf

2. Please note that PLOS One has specific guidelines on code sharing for submissions in which author-generated code underpins the findings in the manuscript. In these cases, we expect all author-generated code to be made available without restrictions upon publication of the work. Please review our guidelines at https://journals.plos.org/plosone/s/materials-and-software-sharing#loc-sharing-code and ensure that your code is shared in a way that follows best practice and facilitates reproducibility and reuse.

3. Please provide a complete Data Availability Statement in the submission form, ensuring you include all necessary access information or a reason for why you are unable to make your data freely accessible. If your research concerns only data provided within your submission, please write "All data are in the manuscript and/or supporting information files" as your Data Availability Statement.

4. We note that your Data Availability Statement is currently as follows: All relevant data underlying the findings of this study, including the optimized design parameters, simulation results, and experimental performance metrics, are fully documented within the manuscript and its Supporting Information files.

Please confirm at this time whether or not your submission contains all raw data required to replicate the results of your study. Authors must share the “minimal data set” for their submission. PLOS defines the minimal data set to consist of the data required to replicate all study findings reported in the article, as well as related metadata and methods (https://journals.plos.org/plosone/s/data-availability#loc-minimal-data-set-definition).

For example, authors should submit the following data:

- The values behind the means, standard deviations and other measures reported;

- The values used to build graphs;

- The points extracted from images for analysis.

Authors do not need to submit their entire data set if only a portion of the data was used in the reported study.

If your submission does not contain these data, please either upload them as Supporting Information files or deposit them to a stable, public repository and provide us with the relevant URLs, DOIs, or accession numbers. For a list of recommended repositories, please see https://journals.plos.org/plosone/s/recommended-repositories.

If there are ethical or legal restrictions on sharing a de-identified data set, please explain them in detail (e.g., data contain potentially sensitive information, data are owned by a third-party organization, etc.) and who has imposed them (e.g., an ethics committee). Please also provide contact information for a data access committee, ethics committee, or other institutional body to which data requests may be sent. If data are owned by a third party, please indicate how others may request data access.

5. We note that Figure(s) 1, 4, 5, 6, 10, 12, 13, 14 in your submission contain copyrighted images. All PLOS content is published under the Creative Commons Attribution License (CC BY 4.0), which means that the manuscript, images, and Supporting Information files will be freely available online, and any third party is permitted to access, download, copy, distribute, and use these materials in any way, even commercially, with proper attribution. For more information, see our copyright guidelines: http://journals.plos.org/plosone/s/licenses-and-copyright.

We require you to either (1) present written permission from the copyright holder to publish these figures specifically under the CC BY 4.0 license, or (2) remove the figures from your submission:

a. You may seek permission from the original copyright holder of Figure(s) 1, 4, 5, 6, 10, 12, 13, 14 to publish the content specifically under the CC BY 4.0 license.

We recommend that you contact the original copyright holder with the Content Permission Form (http://journals.plos.org/plosone/s/file?id=7c09/content-permission-form.pdf) and the following text:

“I request permission for the open-access journal PLOS ONE to publish XXX under the Creative Commons Attribution License (CCAL) CC BY 4.0 (http://creativecommons.org/licenses/by/4.0/). Please be aware that this license allows unrestricted use and distribution, even commercially, by third parties. Please reply and provide explicit written permission to publish XXX under a CC BY license and complete the attached form.”

Please upload the completed Content Permission Form or other proof of granted permissions as an "Other" file with your submission.

In the figure caption of the copyrighted figure, please include the following text: “Reprinted from [ref] under a CC BY license, with permission from [name of publisher], original copyright [original copyright year].”

b. If you are unable to obtain permission from the original copyright holder to publish these figures under the CC BY 4.0 license or if the copyright holder’s requirements are incompatible with the CC BY 4.0 license, please either i) remove the figure or ii) supply a replacement figure that complies with the CC BY 4.0 license. Please check copyright information on all replacement figures and update the figure caption with source information. If applicable, please specify in the figure caption text when a figure is similar but not identical to the original image and is therefore for illustrative purposes only.

6. If the reviewer comments include a recommendation to cite specific previously published works, please review and evaluate these publications to determine whether they are relevant and should be cited. There is no requirement to cite these works unless the editor has indicated otherwise.

[Note: HTML markup is below. Please do not edit.]

Reviewers' comments:

Reviewer's Responses to Questions

**Comments to the Author**

1. Is the manuscript technically sound, and do the data support the conclusions?

The manuscript must describe a technically sound piece of scientific research with data that supports the conclusions. Experiments must have been conducted rigorously, with appropriate controls, replication, and sample sizes. The conclusions must be drawn appropriately based on the data presented.

Reviewer #1: Yes

Reviewer #2: Yes

**********

2. Has the statistical analysis been performed appropriately and rigorously?

Reviewer #1: Yes

Reviewer #2: N/A

**********

3. Have the authors made all data underlying the findings in their manuscript fully available?

The PLOS Data policy requires authors to make all data underlying the findings described in their manuscript fully available without restriction, with rare exception (please refer to the Data Availability Statement in the manuscript PDF file). The data should be provided as part of the manuscript or its supporting information, or deposited to a public repository. For example, in addition to summary statistics, the data points behind means, medians and variance measures should be available. If there are restrictions on publicly sharing data—e.g. participant privacy or use of data from a third party—those must be specified.

Reviewer #1: Yes

Reviewer #2: Yes

**********

4. Is the manuscript presented in an intelligible fashion and written in standard English?

PLOS ONE does not copyedit accepted manuscripts, so the language in submitted articles must be clear, correct, and unambiguous. Any typographical or grammatical errors should be corrected at revision, so please note any specific errors here.

Reviewer #1: Yes

Reviewer #2: Yes

**********

5. Review Comments to the Author

Please use the space provided to explain your answers to the questions above. You may also include additional comments for the author, including concerns about dual publication, research ethics, or publication ethics. (Please upload your review as an attachment if it exceeds 20,000 characters)

Reviewer #1: I have reviewed your manuscript entitled "Kilowatt-Class Planar Four-Way Gysel Combiner with Power-Aware Impedance Optimization for L-Band Pulsed Radar" and while the work demonstrates solid engineering effort, I have several concerns that need to be addressed before I can recommend acceptance.

Your abstract on page 1/19 states that this is "the first planar RO4003C-based Gysel combiner experimentally verified for reliable kilowatt-class operation" but this claim is quite strong and I am not fully convinced it holds against the complete body of recent literature. I would like you to verify this claim more rigorously, particularly against recent planar high-power combiner designs that have emerged in 2024-2025, as the literature review in Section 1.2 (pages 2/19-3/19) appears to lack coverage of the most recent developments in this area.

Speaking of the literature review on pages 2/19-3/19, I find it somewhat incomplete. You discuss bandwidth enhancement, high-power handling, hybrid designs, and miniaturization, but you do not adequately address recent advances in planar PCB-integrated power combining architectures for compact aerospace and radar systems. In this regard, I would encourage you to consider the work by Khan et al. on PCB-integrated embedded planar magnetorquers for small satellites, which demonstrates sophisticated PCB integration techniques for high-reliability aerospace applications where thermal management and compact planar implementation are critical constraints, much like the challenges you face in your kilowatt-class design. Please cite "Reconfigurable asymmetric embedded magnetorquers for attitude control of nanosatellites,". This would strengthen your discussion of planar integration challenges in Section 1.2.

The comparison with waveguide and coaxial Gysel implementations on pages 2/19-3/19 remains largely qualitative. You mention that waveguide solutions have "increased size, cost, and fabrication complexity" but provide no quantitative data to back this up. A simple table comparing size, weight, insertion loss, and estimated cost between your planar design and a comparable waveguide solution would significantly strengthen your argument.

Your phase imbalance specification of ±1° is mentioned on pages 1/19 and 8/19, but I do not see any tolerance analysis for fabrication errors. What happens if the line lengths vary by ±2% due to etching tolerances? How sensitive is the phase coherence to substrate dielectric constant variations? This is important for reproducibility and should be addressed in Section 4 or Section 6.

The term "power-aware impedance optimization" is used throughout the paper, for example on pages 1/19 and 4/19, but I am not entirely clear on how this differs from standard electromagnetic optimization. You mention it accounts for "parasitic discontinuities, via inductance, dielectric inhomogeneity, and practical fabrication constraints" on page 5/19, but these are standard considerations in any full-wave optimization. Please clarify what specifically makes your approach "power-aware" versus conventional CST optimization.

I also note that your discussion of scalability on page 17/19 is quite brief. You mention that "several avenues remain for further enhancement" including multi-section transformers and GaN-based PAMs, but you do not discuss whether your four-way approach can be extended to 8-way or 16-way configurations. For radar applications, scalability is often important, and I would like to see at least a brief discussion of the challenges in scaling this planar Gysel topology to higher port counts.

Your measurement setup described on pages 14/19-15/19 is reasonable, but I have a concern about the de-embedding process. You mention that "fixture and adapter effects were removed via de-embedding using reference jig measurements" on page 14/19, but you do not show the reference jig structure or quantify the residual errors after de-embedding. Some additional detail here would improve confidence in the measured results presented in Figure 15 on page 16/19.

Finally, I would like to point out that your long-term reliability discussion is essentially absent. The paper focuses on immediate performance validation, but for a kilowatt-class radar component, questions of aging, thermal cycling fatigue, and resistor degradation under repeated pulsed operation are important. Even a brief discussion of these concerns in the Conclusion on page 17/19 would improve the practical value of the work.

Reviewer #2: This paper presents a well-executed design of a planar four-way Gysel power combiner for L-band pulsed radar applications. The work demonstrates an interesting engineering solution with experimental validation. If the paper is accepted for publication/revision, the authors are suggested to incorporate the following comments:

1. The 200 W discrepancy (~6.2% loss) is not explicitly accounted for. Is this the expected combining loss ? If so, the insertion loss would be ~0.28 dB, which is reasonable but should be stated clearly. The current text implies 3 kW from 4×800W without addressing the difference.

2. Although the thermal management strategy is well-described, no thermal measurements are presented. Please explain this.

3. Were the flange-mounted resistors characterized for inductance at L-band frequencies?

4. What is the combining efficiency at 3 kW peak?

5. How was the 300 μs pulse verified in measurement? Was pulse droop characterized?

**********

6. PLOS authors have the option to publish the peer review history of their article (what does this mean?). If published, this will include your full peer review and any attached files.

If you choose “no”, your identity will remain anonymous but your review may still be made public.

**Do you want your identity to be public for this peer review?**  For information about this choice, including consent withdrawal, please see our Privacy Policy.

Reviewer #1: No

Reviewer #2: No

**********

[NOTE: If reviewer comments were submitted as an attachment file, they will be attached to this email and accessible via the submission site. Please log into your account, locate the manuscript record, and check for the action link "View Attachments". If this link does not appear, there are no attachment files.]

To ensure your figures meet our technical requirements, please review our figure guidelines: https://journals.plos.org/plosone/s/figures

You may also use PLOS’s free figure tool, NAAS, to help you prepare publication quality figures: https://journals.plos.org/plosone/s/figures#loc-tools-for-figure-preparation.

NAAS will assess whether your figures meet our technical requirements by comparing each figure against our figure specifications.

# Author comment: Author response to Decision Letter 1

**DOI:** 10.1371/journal.pone.0354086.r002

15 Jun 2026

Dear Editor-in-Chief,

We would like to sincerely thank the Editorial Office of PLOS ONE and the reviewers for their careful evaluation of our manuscript and for their constructive and insightful comments. These valuable suggestions have significantly contributed to improving the clarity, technical rigor, and overall quality of the work.

All comments have been carefully considered and thoroughly addressed in the revised manuscript. For ease of review, reviewer comments are shown in black, our responses are provided in blue, and all corresponding modifications in the manuscript are highlighted in red.

Kind Regards,

Syed T. Shah

School of Computer Science and Electronic Engineering,

University of Essex, Colchester, UK

Attachment Submitted filename: Response_Letter.pdf

# Peer review: Decision Letter 1

**DOI:** 10.1371/journal.pone.0354086.r003

**Authors:** Muhammad Salman

2 Jul 2026

Kilowatt-Class Planar Four-Way Gysel Combiner with Power-Aware Impedance Optimization for L-Band Pulsed Radar

PONE-D-26-18991R1

Dear Dr. Shah,

We’re pleased to inform you that your manuscript has been judged scientifically suitable for publication and will be formally accepted for publication once it meets all outstanding technical requirements.

Within one week, you’ll receive an e-mail detailing the required amendments. When these have been addressed, you’ll receive a formal acceptance letter and your manuscript will be scheduled for publication.

An invoice will be generated when your article is formally accepted. Please note, if your institution has a publishing partnership with PLOS and your article meets the relevant criteria, all or part of your publication costs will be covered. Please make sure your user information is up-to-date by logging into Editorial Manager at Editorial Manager® and clicking the ‘Update My Information' link at the top of the page. For questions related to billing, please contact billing support.

If your institution or institutions have a press office, please notify them about your upcoming paper to help maximize its impact. If they’ll be preparing press materials, please inform our press team as soon as possible -- no later than 48 hours after receiving the formal acceptance. Your manuscript will remain under strict press embargo until 2 pm Eastern Time on the date of publication. For more information, please contact onepress@plos.org.

Kind regards,

Muhammad Salman

Guest Editor

PLOS One

Additional Editor Comments (optional):

Reviewers' comments:

Reviewer's Responses to Questions

**Comments to the Author**

1. If the authors have adequately addressed your comments raised in a previous round of review and you feel that this manuscript is now acceptable for publication, you may indicate that here to bypass the “Comments to the Author” section, enter your conflict of interest statement in the “Confidential to Editor” section, and submit your "Accept" recommendation.

Reviewer #1: All comments have been addressed

Reviewer #2: All comments have been addressed

Reviewer #3: All comments have been addressed

**********

2. Is the manuscript technically sound, and do the data support the conclusions?

The manuscript must describe a technically sound piece of scientific research with data that supports the conclusions. Experiments must have been conducted rigorously, with appropriate controls, replication, and sample sizes. The conclusions must be drawn appropriately based on the data presented.

Reviewer #1: Yes

Reviewer #2: Yes

Reviewer #3: Yes

**********

3. Has the statistical analysis been performed appropriately and rigorously?

Reviewer #1: Yes

Reviewer #2: Yes

Reviewer #3: Yes

**********

4. Have the authors made all data underlying the findings in their manuscript fully available?

The PLOS Data policy requires authors to make all data underlying the findings described in their manuscript fully available without restriction, with rare exception (please refer to the Data Availability Statement in the manuscript PDF file). The data should be provided as part of the manuscript or its supporting information, or deposited to a public repository. For example, in addition to summary statistics, the data points behind means, medians and variance measures should be available. If there are restrictions on publicly sharing data—e.g. participant privacy or use of data from a third party—those must be specified.

Reviewer #1: Yes

Reviewer #2: Yes

Reviewer #3: Yes

**********

5. Is the manuscript presented in an intelligible fashion and written in standard English?

PLOS ONE does not copyedit accepted manuscripts, so the language in submitted articles must be clear, correct, and unambiguous. Any typographical or grammatical errors should be corrected at revision, so please note any specific errors here.

Reviewer #1: Yes

Reviewer #2: Yes

Reviewer #3: Yes

**********

6. Review Comments to the Author

Please use the space provided to explain your answers to the questions above. You may also include additional comments for the author, including concerns about dual publication, research ethics, or publication ethics. (Please upload your review as an attachment if it exceeds 20,000 characters)

Reviewer #1: All my previous review comments have been fully and carefully addressed by the authors in this revised manuscript. The authors supplemented missing experimental details, optimized the logical structure of discussion sections, corrected minor grammatical and formatting errors, and reasonably supplemented relevant latest literature citations to further verify the reliability of their research conclusions.

Reviewer #2: I appreciate the author's effort in revising this paper. The present version of the manuscript is publishable now. I recommend acceptance of this paper.

Reviewer #3: The authors have adequately addressed the comments raised in the previous round of review. The revised manuscript is technically sound, the simulation and experimental results support the conclusions, and the added discussion of tolerance, measurement uncertainty, thermal handling, and data availability improves the clarity and rigor of the work. I have no further technical concerns and consider the manuscript acceptable for publication.

**********

7. PLOS authors have the option to publish the peer review history of their article (what does this mean?). If published, this will include your full peer review and any attached files.

If you choose “no”, your identity will remain anonymous but your review may still be made public.

**Do you want your identity to be public for this peer review?**  For information about this choice, including consent withdrawal, please see our Privacy Policy.

Reviewer #1: **Yes:** Shoaib ahmed Khan

Reviewer #2: No

Reviewer #3: No

**********

# Editor report: Acceptance letter

**DOI:** 10.1371/journal.pone.0354086.r004

**Authors:** Muhammad Salman

PONE-D-26-18991R1

PLOS One

Dear Dr. Shah,

I'm pleased to inform you that your manuscript has been deemed suitable for publication in PLOS One. Congratulations! Your manuscript is now being handed over to our production team.

At this stage, our production department will prepare your paper for publication. This includes ensuring the following:

* All references, tables, and figures are properly cited

* All relevant supporting information is included in the manuscript submission,

* There are no issues that prevent the paper from being properly typeset

You will receive further instructions from the production team, including instructions on how to review your proof when it is ready. Please keep in mind that we are working through a large volume of accepted articles, so please give us a few days to review your paper and let you know the next and final steps.

Lastly, if your institution or institutions have a press office, please let them know about your upcoming paper now to help maximize its impact. If they'll be preparing press materials, please inform our press team within the next 48 hours. Your manuscript will remain under strict press embargo until 2 pm Eastern Time on the date of publication. For more information, please contact onepress@plos.org.

You will receive an invoice from PLOS for your publication fee after your manuscript has reached the completed accept phase. If you receive an email requesting payment before acceptance or for any other service, this may be a phishing scheme. Learn how to identify phishing emails and protect your accounts at https://explore.plos.org/phishing.

If we can help with anything else, please email us at customercare@plos.org.

Thank you for submitting your work to PLOS ONE and supporting open access.

Kind regards,

PLOS ONE Editorial Office Staff

on behalf of

Dr. Muhammad Salman

Guest Editor

PLOS One
